# Integrated Bioinformatics Analysis of Shared Genes, miRNA, Biological Pathways and Their Potential Role as Therapeutic Targets in Huntington’s Disease Stages

**DOI:** 10.3390/ijms24054873

**Published:** 2023-03-02

**Authors:** Christiana C. Christodoulou, Eleni Zamba Papanicolaou

**Affiliations:** Neuroepidemiology Department, The Cyprus Institute of Neurology and Genetics, 2371 Nicosia, Cyprus

**Keywords:** Huntington’s Disease, molecular mechanisms, bioinformatics approaches, monogenic disease, neurodegenerative disease

## Abstract

Huntington’s Disease (HD) is a progressive neurodegenerative disease caused by CAG repeat expansion in the huntingtin gene (HTT). The HTT gene was the first disease-associated gene mapped to a chromosome, but the pathophysiological mechanisms, genes, proteins or miRNAs involved in HD remain poorly understood. Systems bioinformatics approaches can divulge the synergistic relationships of multiple omics data and their integration, and thus provide a holistic approach to understanding diseases. The purpose of this study was to identify the differentially expressed genes (DEGs), HD-related gene targets, pathways and miRNAs in HD and, more specifically, between the pre-symptomatic and symptomatic HD stages. Three publicly available HD datasets were analysed to obtain DEGs for each HD stage from each dataset. In addition, three databases were used to obtain HD-related gene targets. The shared gene targets between the three public databases were compared, and clustering analysis was performed on the common shared genes. Enrichment analysis was performed on (i) DEGs identified for each HD stage in each dataset, (ii) gene targets from the public databases and (iii) the clustering analysis results. Furthermore, the hub genes shared between the public databases and the HD DEGs were identified, and topological network parameters were applied. Identification of HD-related miRNAs and their gene targets was obtained, and a miRNA-gene network was constructed. Enriched pathways identified for the 128 common genes revealed pathways linked to multiple neurodegeneration diseases (HD, Parkinson’s disease, Spinocerebellar ataxia), MAPK and HIF-1 signalling pathways. Eighteen HD-related hub genes were identified based on network topological analysis of MCC, degree and closeness. The highest-ranked genes were *FoxO3* and *CASP3*, *CASP3* and *MAP2* were found for betweenness and eccentricity and *CREBBP* and *PPARGC1A* were identified for the clustering coefficient. The miRNA-gene network identified eleven miRNAs (mir-19a-3p, mir-34b-3p, mir-128-5p, mir-196a-5p, mir-34a-5p, mir-338-3p, mir-23a-3p and mir-214-3p) and eight genes (*ITPR1*, *CASP3*, *GRIN2A*, *FoxO3*, *TGM2*, *CREBBP, MTHFR* and *PPARGC1A*). Our work revealed that various biological pathways seem to be involved in HD either during the pre-symptomatic or symptomatic stages of HD. This may offer some clues for the molecular mechanisms, pathways and cellular components underlying HD and how these may act as potential therapeutic targets for HD.

## 1. Introduction

Huntington’s Disease (HD) was initially presented and explained in 1872 by Dr. George Huntington [1]; it is a rare and progressive neurodegenerative disease passing within families from generation to generation in an autosomal dominant mode of inheritance [1]. HD affects the central nervous system and, more specifically, the medium spiny neurons (MSN) of the basal ganglia [2], resulting in unwanted choreatic movements and cognitive and behavioural impairment [1].

HD is largely caused by an inherited CAG trinucleotide repeat expansion on the huntingtin (HTT) gene that is located on exon 1 of chromosome 4 [3,4]. The huntingtin protein (HTT) is encoded by the *HTT* gene [3]. The HTT protein is involved in various functions, including neural tube formation, axonal transport, synaptic function, transcription, immune and mitochondrial function and cell survival [5]. The *HTT* gene is located in a repeated DNA fragment consisting of cytosine-adenine-guanine (CAG), which are repeated multiple times resulting in mutant HTT (mHTT), which leads to a disruption of numerous cellular functions and eventually neuronal cell death [3,5].

The number of CAG repeat expansions is the main predictor for age of onset and disease severity in HD [3]. Repeat length between 10 and 35 CAG units is within the normal range. Repeat length between 36 and 39 CAG units confers reduced penetrance, where some individuals may or may not develop the disease within their lifespan. Repeats of 40 or more CAG units are pathogenic, resulting in the development of signs and symptoms of HD [4,5]. In addition, the longer the CAG repeats, the earlier the age of onset of HD [5].

The typical age of onset for HD is approximately 40 years, with an average life expectancy of 17 years after symptom onset [2]. Clinical characteristics include (i) movement impairment such as choreatic movements and incoordination, (ii) cognitive impairment such as a lapse in short-term memory and (iii) behavioural impairment such as depression, personality changes, suicidal thoughts and psychosis [1,4]. The clinical characteristics of HD patients are assessed using the Unified Huntington’s Disease Rating Scale (UHDRS), which evaluates the domains of (i) motor function, (ii) cognition, (iii) behaviour and (iv) functional abilities of pre-symptomatic and symptomatic HD patients [1].

HD is a rare monogenic and incurable disease that involves a complex web of pathogenic mechanisms affecting multiple cellular processes, genes, proteins, metabolites and several additional biological entities.

These disease components need to be further investigated in order to understand HD pathology, with the aim to develop treatments that not only target disease symptoms, such as movement, cognitive and behavioural impairment, but also target the causative mutation at the DNA and RNA level [5].

Systems bioinformatics (SB) allows us to obtain a better grasp of the disease pathophysiology by integrating different biological omics data to better understand the molecular pathways, mechanisms, genes, microRNAs (miRNAs), proteins and metabolites involved in HD and lead to possible therapeutic drug treatments and the discovery of biomarkers [6].

The main approach in this direction is the generation and construction of biological networks representing each level of omics data and their integration into a multi-layered network that allows the exchange of information within and between layers. Here, the goal is to reveal synergistic relationships among various biological entities rather to explore each one individually [6]. The evolving importance of biological network-based approaches allows for the development of potential biological and clinical applications by providing an intuitive approach to explore the biological and molecular complexity of not only neurodegenerative diseases, but any disease of interest [7].

The purpose of this study is to integrate and analyse gene data and miRNAs related to HD pathogenesis from publicly available databases, which can provide further insight into the molecular mechanisms involved in HD through integrated bioinformatics analysis, which can be used to develop dual-purpose prevention methods and promising clues for further experimental studies, pharmacological networks, novel treatments, drug discovery and repurposing and biomarker discovery.

## 2. Results

### 2.1. GEO Dataset Information

Three transcriptomics datasets with accession numbers of GSE1751 [8], GSE24250 [9] and GSE135589 [10], respectively, were selected to be included in this study. For detailed description of the GEO datasets, see Table 1.

### 2.2. Differentially Expressed Genes of the HD-Related Datasets

The top 200 over- and top 200 under-expressed differentially expressed genes (DEGs), therefore a total of 400 genes were identified respectively for each HD-related dataset. The DEGs for: (i) the pre-symptomatic (PreHD) and symptomatic HD (SymHD) patients in comparison to controls (GSE1751), (ii) HD patients versus controls (GSE24250), (iii) Stage 1 HD patients (Year 1) versus controls and (iv) Stage 2 HD patients (Year 2) versus controls (GSE135589), are shown in the Supplementary Materials (Tables S1–S5, respectively).

### 2.3. Gene Co-Expression Networks of HD-Related Datasets

**Figure 1:**
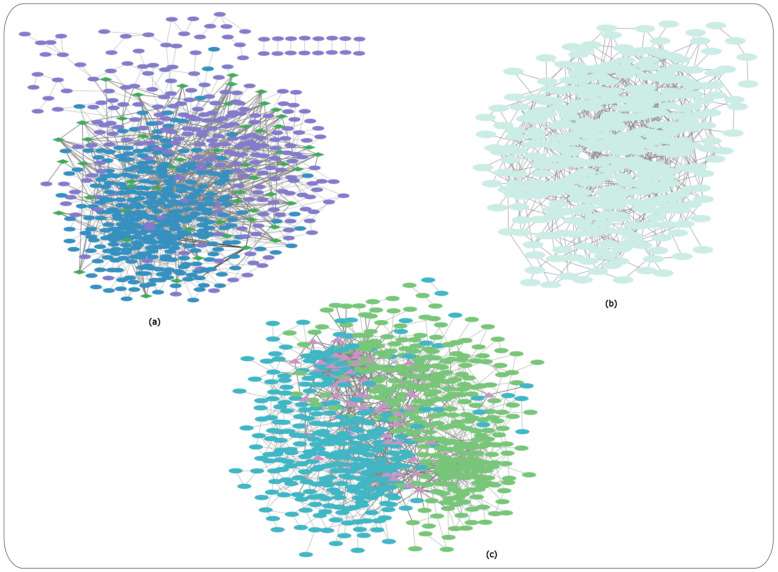
Gene co-expression networks for each of the HD-related datasets. (**a**) Controls versus pre-symptomatic HD patients and controls versus symptomatic HD patients, blue-coloured: genes present in controls versus pre-symptomatic HD patients, purple-coloured: genes present in controls versus symptomatic HD patients, green-coloured diamond nodes: genes present in both pre-symptomatic and symptomatic HD patients; (**b**) Co-expression network for controls versus HD patients; (**c**) Controls and Stage 1 and 2 HD patients (Year 1 and 3), blue-coloured nodes: genes present in controls versus Stage 1 (Year 1) HD patients, green-coloured nodes: genes present in controls versus Stage 2 (Year 3) HD patients, purple-coloured triangular nodes: genes present in both Stage 1 and Stage 2 HD patients.

The gene co-expression networks containing the DEGs for each HD dataset as mentioned above are shown in (Figure 1a–c), respectively. The genes are represented as nodes and the edges represent the co-expression among the genes between controls versus HD patients. The three co-expression networks were created and visualized using the Cytoscape software [11].

In the case of controls versus PreHD patients and controls versus SymHD patients, the blue-coloured nodes represent genes present in controls versus PreHD patients and purple-coloured nodes represent genes present in controls versus SymHD patients. Meanwhile, green-coloured diamond nodes represent the genes present in both pre-symptomatic and symptomatic HD patients (Figure 1a). Table 2 indicates the 54 shared genes between the merged networks in PreHD and SymHD patients versus controls. The gene co-expression network for controls versus HD patients (Figure 1b).

In the case of controls and Stage 1 HD patients (Year 1), the blue-coloured nodes represent the genes present in controls versus Stage 1 (Year 1) HD patients, and green-coloured nodes represent the genes present in controls versus Stage 2 (Year 3) HD patients, whereas the purple-coloured triangular nodes present the genes present in both Stage 1 and Stage 2 HD patients (Figure 1c). Table 3 indicates the 70 shared genes between the merged networks in Stage 1 (Year 1) and Stage 2 (Year 3) HD patients versus controls.

**Figure 2:**
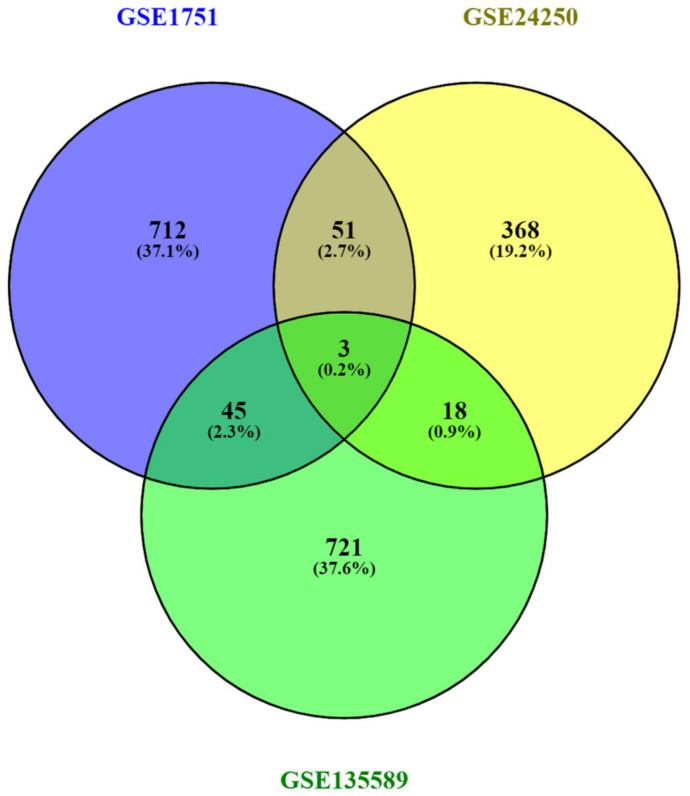
Venn diagram representing the common and exclusive DEGs between the GSE1751, GSE24250 and GSE135589 datasets.

Furthermore, the intersection of the Venn (https://bioinfogp.cnb.csic.es/tools/venny/ accessed on 12 December 2022) diagram was used to identify the common DEGs (cDEGs) shared between the three datasets of GSE1751, GSE24250 and GSE135589 (Figure 2). A total of three cDEGs were identified to be shared among the three datasets: GP1BB, SEPT5 and SEPT5-GP1BB.

### 2.4. Metascape Enrichment Analysis of DEGs for Each HD-Related Dataset

Enrichment analysis [12] used the Kyoto Encyclopaedia of Genes and Genomes (KEGG) and Gene Ontology (GO) libraries of biological processes, cellular components and molecular function. The top 200 over- and top 200 under-expressed DEGs for each HD-related dataset (GSE1751, GSE24250 and GSE 135589) were used as input.

**Figure 3:**
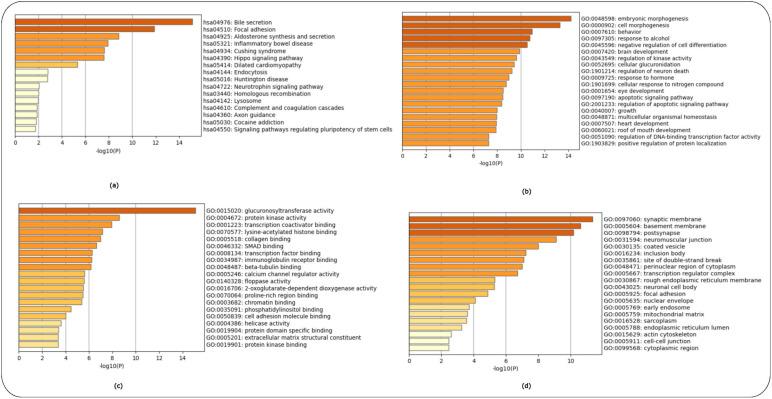
Enrichment analysis of controls versus pre-symptomatic HD patients using the KEGG and GO libraries in Metascape. (**a**) KEGG pathways, (**b**) GO Biological Process, (**c**) GO Molecular Function and (**d**) GO Cellular Components.

**Figure 4:**
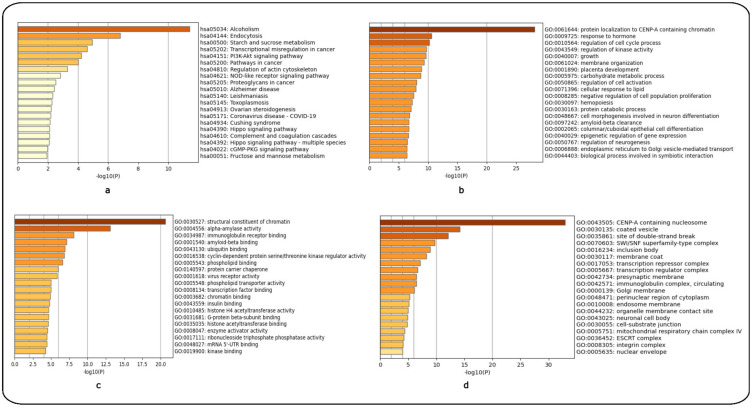
Enrichment analysis of controls versus symptomatic HD patients using the KEGG and GO libraries in Metascape. (**a**) KEGG pathways, (**b**) GO Biological Process, (**c**) GO Molecular Function and (**d**) GO Cellular Components.

Enrichment analysis for controls versus pre-symptomatic and controls versus symptomatic HD patients (GSE1751) used the top 200 over- and 200 under-expressed DEGS identified for each HD stage. The top-15 ranked scoring pathways were identified for each HD stage for the GSE1751 dataset for the KEGG as Table S6 and for the GO terms of biological processes, molecular function and cellular components as (Tables S7–S9), respectively, and the enrichment graphs as (Figure 3a–d and Figure 4a–d), respectively, for each HD stage.

**Figure 5:**
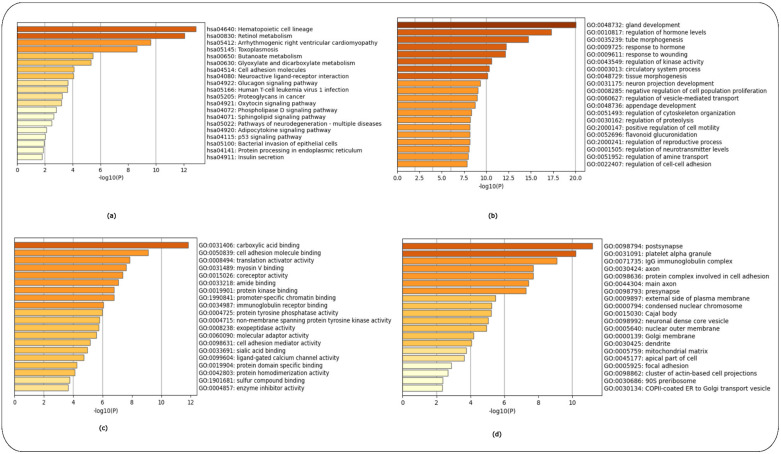
Enrichment analysis of controls versus HD patients using the KEGG and GO libraries in Metascape. (**a**) KEGG pathways, (**b**) GO Biological Process, (**c**) GO Molecular Function and (**d**) GO Cellular Components.

The enrichment analysis for controls versus HD patients was performed using the top 200 over- and 200 under-expressed DEGs. The top-15 ranked scoring pathways identified for controls versus HD patients for the GSE24250 dataset using the KEGG (Table S10) and GO libraries (Table S11), and the related enrichment graphs are shown in (Figure 5a–d).

**Figure 6:**
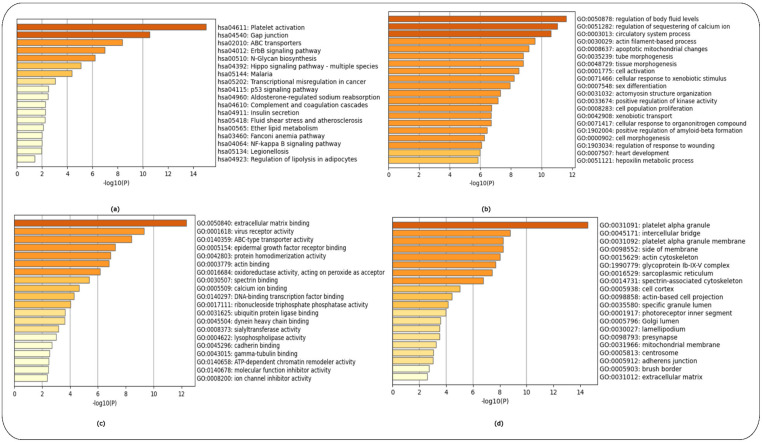
Enrichment analysis of controls versus Stage 1 HD patients (Year 1) using the KEGG and GO libraries in Metascape. (**a**) KEGG pathways, (**b**) GO Biological Process, (**c**) GO Molecular Function and (**d**) GO Cellular Components.

**Figure 7:**
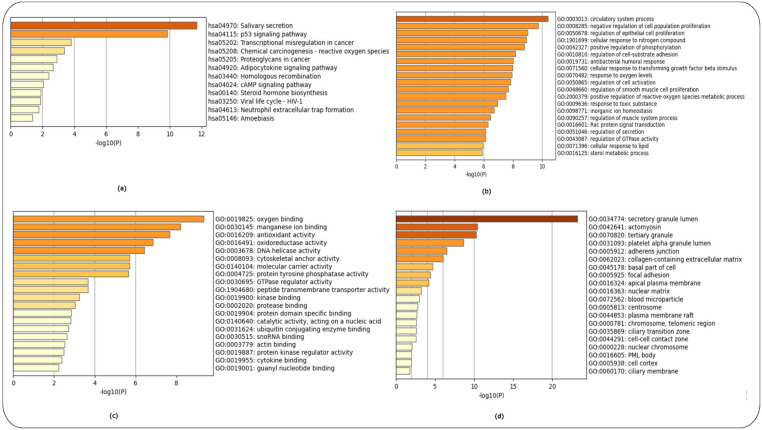
Enrichment analysis of controls versus Stage 2 HD patients (Year 3) using the KEGG and GO libraries in Metascape. (**a**) KEGG pathways, (**b**) GO Biological Process, (**c**) GO Molecular Function and (**d**) GO Cellular Components.

Lastly, enrichment analysis was performed for controls versus Stage 1 (Year 1) HD patients and controls versus Stage 2 (Year 3) using the top 200 over- and 200 under-expressed DEGs identified for each HD stage. The top-15 ranked scoring pathways identified for each HD stage for the GSE135589 dataset are shown using the KEGG (Table S12) and GO libraries (Table S13) for Stage 1 (Year 1). Table S14 shows the top-12ranked scoring pathways identified using the KEGG library, while Table S15 shows the top-15 ranked scoring pathways using the GO libraries (see Table S15) for Stage 2 (Year 3); for the related enrichment graphs see (Figure 6a–d and Figure 7a–d), respectively.

### 2.5. Analysis of Common Gene Targets from Three Public Databases

**Figure 8:**
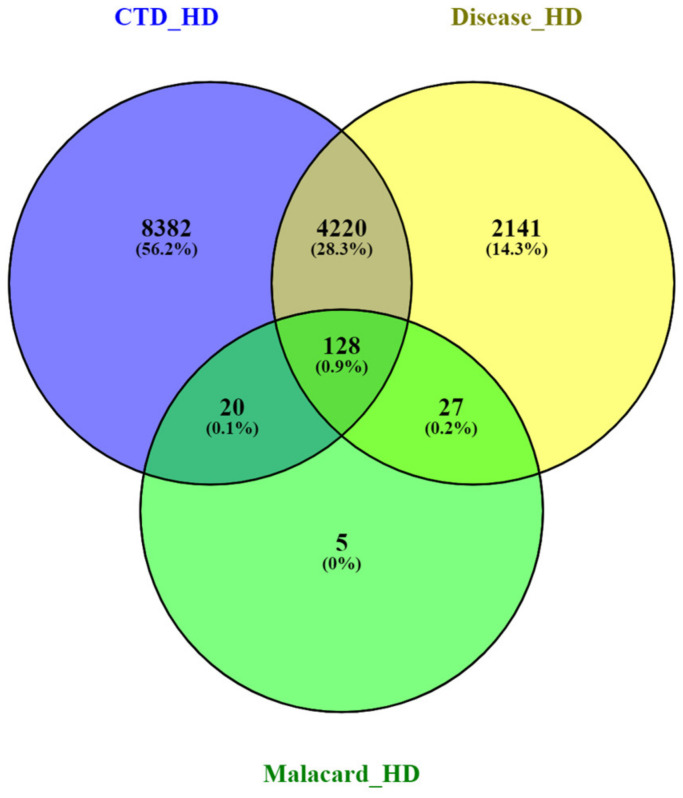
Venn diagram representing the common and exclusive gene targets of the HD CTD, Disease and Malacards databases.

In order to integrate the biological data, we combined the HD-related genes available in the CTD, Malacards and Diseases databases using the intersection of a Venn diagram (https://bioinfogp.cnb.csic.es/tools/venny/ accessed on 27 December 2022). A total of 128 common gene targets were found to be shared between the three databases (Figure 8). The list of the 128 common gene targets of HD can be found in the Supplementary Materials as (Table S16).

**Figure 9:**
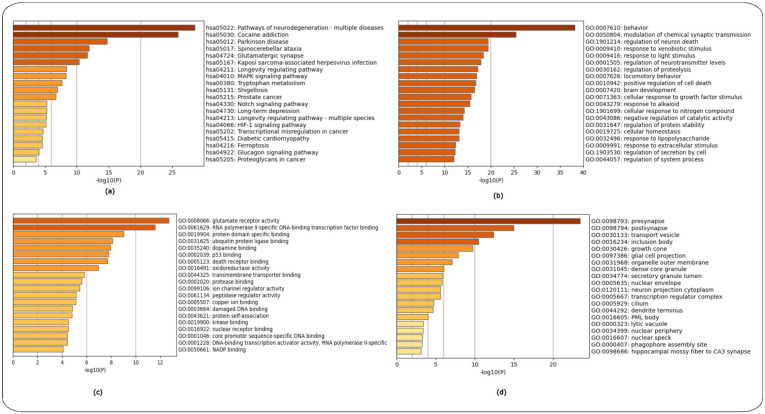
Enrichment analysis of gene targets shared between the public databases of CTD, Malacards and Disease Database using the KEGG and GO libraries in Metascape. (**a**) KEGG pathways, (**b**) GO-Biological Process, (**c**) GO-Molecular Function and (**d**) GO-Cellular Components.

Enrichment analysis using the KEGG and GO libraries was performed on the 128 common gene targets using Metascape. The enrichment graphs are shown as (Figure 9). KEGG enrichment analysis of the 128 common genes revealed that most of the shared gene-related pathways are linked to pathways involved in cocaine addiction and pathways of multiple neurodegeneration diseases, including HD, Parkinson’s disease and Spinocerebellar ataxia. Furthermore, there are a number of signalling pathways involved such as MAPK signalling, HIF-1 signalling and Notch signalling pathways. Metabolism pathways were also found to be enriched, more specifically the tryptophan metabolism pathway (Figure 9a).

The GO biological processes revealed that most of the shared gene-related pathways are linked to behaviour, modulation of chemical synaptic transmission, regulation of neuron death, response to xenobiotic stimulus, response to extracellular stimulus and response to oxygen levels (Figure 9b).

The GO molecular function revealed pathways linked to glutamate receptor activity, RNS polymerase II specific DNA binding transcription factor binding, p53 binding, death receptors, dopamine, ubiquitin protein ligase binding, nuclear receptor binding and protease binding, along with several additional pathways (Figure 9c).

The GO cellular components revealed pathways linked mainly to the nervous system including pre-synapse, cell body, inclusion body, neuron to neuron synapse GABA-ergic synapse and various additional cellular components linked to the nervous system (Figure 9d).

**Figure 10:**
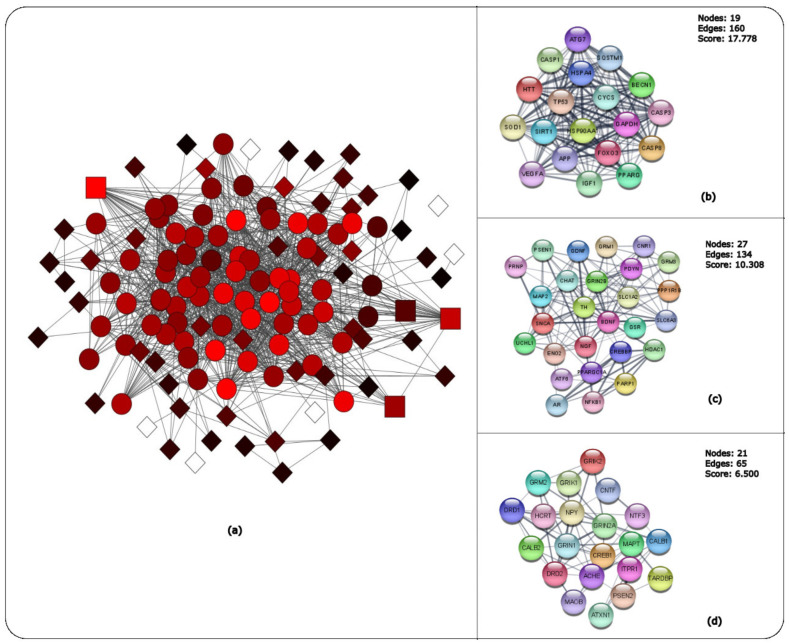
MCODE Cluster analysis of the 128 common genes between the three public databases. (**a**) The PPI of the 128 common genes visualized and analysed in MCODE, highlighting the three clusters. Square nodes represent seeds with the highest score, circle nodes represent clustered nodes and diamond nodes represent unclustered node. Black to red MCODE nodes represent lowest- to highest-scoring nodes and white-coloured nodes represent zero MCODE score. Subnetworks indicating (**b**) cluster 1 (**c**) cluster 2 and (**d**) cluster 3 components.

A protein-to-protein interaction (PPI) network of the 128 common gene targets was constructed and visualized in STRING with combined scores greater than 0.4. The combined score is computed by integrating the probabilities from the numerous different types of evidence (gene fusion, co-occurrence, co-expression, experimental, text mining and conserved neighborhood), while correcting for the probability of randomly observing an interaction [13]. The PPI network was then imported to Cytoscape [11] for further analysis. The PPI network consists of 127 nodes and 1202 edges (Figure 10a). MCODE [14] used to identify the closely connected gene modules within the PPI network. Three closely connected gene modules were identified via the MCODE plug-in of Cytoscape (Figure 10b–d and Table S17). The highest-ranking cluster was cluster 1 with a score of 17.7, followed by cluster 2 with a score of 10.3 and cluster 3 with a score of 6.5. The top-ranked gene targets for each cluster can be found in the Supplementary Materials (Tables S18 and S19). In addition, enrichment analysis was performed for each of the gene clusters for KEGG (Figure S1) and GO (Figures S2–S4) analysis.

The enrichment analysis results identified neurodegenerative pathways, such as HD, Alzheimer’s Disease and PD, and signalling pathways, such as cAMP, Rap1 and calcium signalling. Some of the GO biological processes obtained include regulation to oxidative stress, pathways related to neuronal cell death (regulation of apoptotic signalling pathways, positive regulation of apoptotic process and regulation of neuron death) and pathways involved in the central nervous system (CNS) (negative regulation of synaptic transmission, regulation of long-term synaptic potentiation and trans-synaptic signalling). Meanwhile, the GO molecular functions for all three clusters focused on glutamate receptor activity, calcium channel regulator activity, ubiquitin protein ligase binding and several GO terms. The GO cellular components that were pre-dominantly significant were GO terms that were involved in synaptic functions or neuronal structures (axons, synaptic membranes, synaptic vesicles and dendrites). The following pathways can be found in the Supplementary Materials (Figures S1 and S4, respectively).

### 2.6. HD-Related Hub Gene Identification

**Figure 11:**
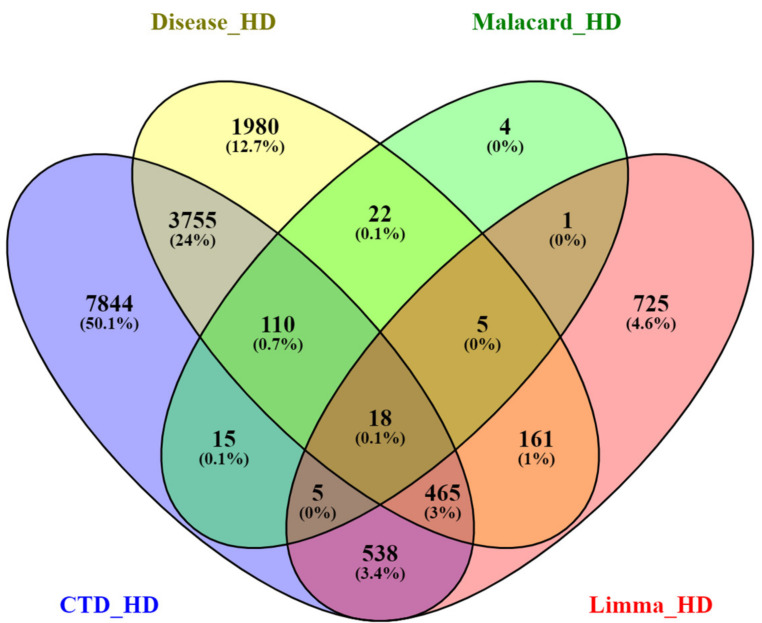
Venn diagram representing the common and exclusive gene targets of the HD CTD, Disease and Malacard databases and all DEGs identified from the HD-related datasets.

The intersection of the Venn diagram (Figure 11) revealed a total of 18 hub genes (Table 4) that are shared between the three public databases and the DEGs identified via Limma for all three HD datasets.

**Figure 12:**
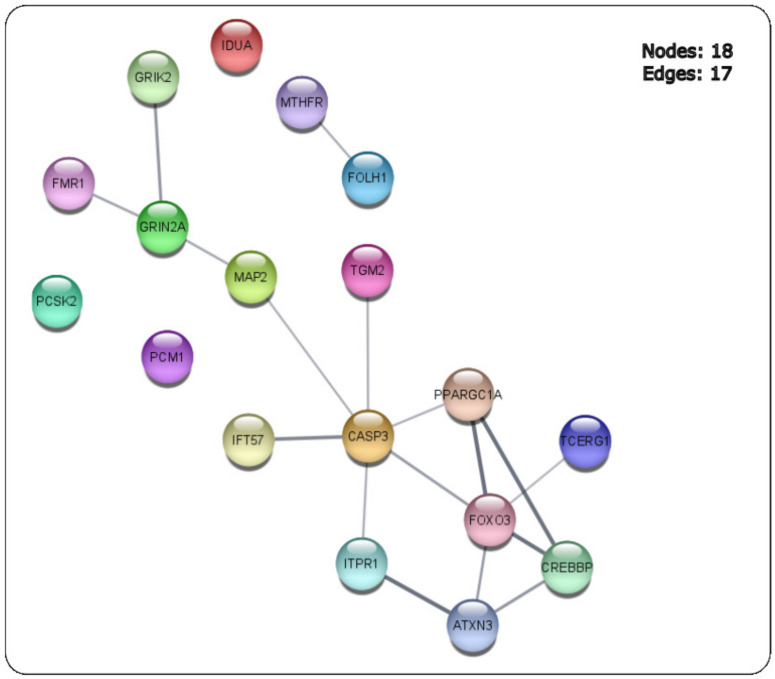
PPI network of the 18 HD-related hub genes identified to be shared between the public databases and the HD DEGs between the three GEO datasets.

**Figure 13:**
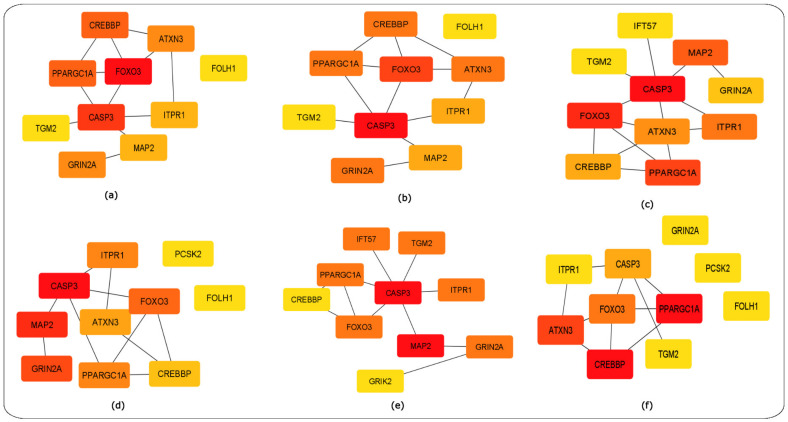
CytoHubba analysis results of the 18 HD-related hub genes. Red- to yellow-coloured CytoHubba nodes represent highest- to lowest-ranked nodes based on the topological approach applied. (**a**) MCC ranking method, (**b**) degree ranking method, (**c**) closeness ranking method, (**d**) betweenness ranking method (**e**) eccentricity ranking method and (**f**) clustering coefficient ranking method.

Networks are a useful approach to visualize various types of biological data such as PPI, gene regulation, metabolite, cellular pathways and signal transduction pathways [15]. The 18 HD-related hub genes were first mapped in STRING [13] (Figure 12) with the medium confidence score of the PPI set at 0.400. CytoHubba was used to perform network topological measures on the 18 HD-related hub genes using the parameters of (i) MCC, (ii) degree, (iii) closeness, (iv) betweenness, (v) eccentricity and (vi) clustering coefficient [11,15]. The ranked gene networks using each of the network parameters are shown as (Figure 13a–f).

The topological network parameters of MCC, degree and closeness identified the highest-ranked genes to be *FoxO3* and *CASP3*. Meanwhile, the betweenness and eccentricity approaches identified the highest-ranked genes of *CASP3* and *MAP2*, and the genes of *CREBBP* and *PPARGC1A* were highly ranked for the clustering coefficient network measure. For the six network scoring measures for each of the top-10 ranked genes (Table 5).

### 2.7. Identification and Analysis of Common miRNAs in HD

A total of 20 miRNAs associated with HD were screened and identified from the Human microRNA Disease database (HMDD) (Table S20). Based on the literature, referencing the HMDD, the following HD-related miRNAs were identified for each of the subsequent categories: (i) HD pathogenic (miR-10-5p), (ii) decreased gene expression (miR-146a, mirR-125b, miR-125b-2a), (iii) increased expression (miR-214), (iv) targeting of HD gene (miR-214, mirR-150, miR-146a, miR-125b and miR-34a) (v) biomarkers (miR-338-3p, miR-128-3p, miR-23a-3p, miR-24-3p, miR-22 and miR-34b), (vi) therapeutic markers (miR-128a, miR-132, miR-196a, miR-200a and miR-200c), (vii) functional roles (miR-137, miR214 and miR-148a), (viii) regulation of HTT (miR-137, miR-124 and miR-148a and ix) other (miR-137). The miRDB tool [16,17] identified the 5p or the 3p of the HD-related miRNAs, as most of the miRNAs were too ambiguous to perform enrichment analysis.

Enrichment analysis using the KEGG library of the mirPath v.3 tool [18,19] was performed for the 20 HD miRNAs. However, the miRNAs of miR-128-5p, miR-137-5p and miR-137-3p were not included in the enrichment analysis, as they are not in the mirPath database.

The KEGG enrichment results identified several signalling pathways such as the ErbB, TGF-beta, neurotrophin signalling, Foxo, MAPK, cAMP, Hippo and PI3K-Akt signalling pathways. In addition, various pathways involved in nervous system functions, such as long-term potentiation, SNARE interactions in vesicular transport and axon guidance, were also found. Pathways with *p*-values <0.05 were considered to be statistically significant. The KEGG pathways and the miRNAs identified can be found in the Supplementary Materials (Table S21).

### 2.8. The Common miRNAs-Gene Network

**Figure 14:**
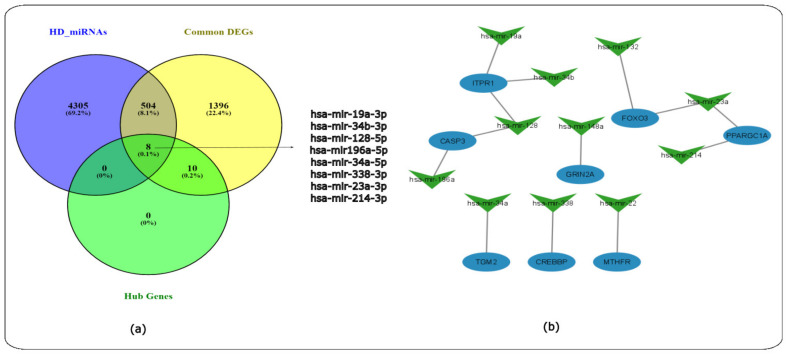
Venn diagram and miRNA-gene network for HD. (**a**) Venn diagram indicating the miRNA gene targets, the DEGs identified for all the HD datasets and the 18 hub genes, (**b**) miRNA-gene network where green V-shaped nodes represent the miRNAs and the blue circular nodes represent the eight common genes.

A total of 7027 target genes for the twenty common miRNAs were predicted using miRTarbase [20]. The HD miRNA-gene network was constructed by taking the intersection of the Venn diagram for the miRNA gene targets obtained via miRTarbase, the HD DEGs identified for all HD datasets and the 18 hub genes identified (Figure 14a). A total of eight common genes were identified to be shared between HD miRNAs, HD DEGs and the 18 hub genes. The miRNA-gene network was constructed and visualized in Cytoscape [11]. The miRNA-gene network contains 11 miRNAs as indicated by the green V-shaped nodes, and the eight common shared nodes are indicated as the blue-coloured circular nodes (Figure 14b).

The KEGG enrichment analysis of these miRNAs using miRPath [18,19] identified signalling pathways, such as the ErbB, Rap1, Ras, TGF-beta and other signalling pathways. In addition, pathways involved in the nervous system such as SNARE interactions in vesicular transport, long-term potentiation and the synaptic vesicle cycle were also identified. The KEGG pathways identified can be found in the Supplementary Materials (Table S22). Therefore, the following 11 miRNAs may play a role in signalling and nervous system pathways that become dysfunctional in HD.

## 3. Discussion

HD is a rare monogenic neurodegenerative disease characterized by a CAG repeat expansion in the *HTT* gene that results in motor, cognitive and behavioural impairment in HD-affected individuals. The molecular mechanisms contributing to HD pathology remain highly complex, making pharmacology and therapeutic intervention difficult, which has resulted in various HD clinical trials being halted, either due to safety concerns or no observable changes between the HD stages of pre-symptomatic and symptomatic HD patients [3,21]. The utilization of several bioinformatics approaches and tools provides a more holistic understanding of the genes, molecular mechanisms and interactions of miRNA genes involved in HD. The aim of this study to was investigate the genes, pathways and miRNAs in HD and, more specifically, between the HD stages of pre-symptomatic and symptomatic HD by combining various types of data ranging from gene expression and biological pathways and mechanisms to HD gene targets and miRNA from public databases in order to shed light on potential biological entities that may act as novel pharmacological targets for treatment of the different HD stages. Some of the KEGG HD-related pathways identified to be common between the three HD datasets of GSE1751, GSE24250 and GSE135589 include the Hippo, neurotrophin, PI3K-Akt and p53 signalling pathways. The complement and coagulation cascade and multiple neurodegenerative pathways have also been observed to be shared among the datasets.

In regards to the GO biological process, numerous GO terms that play a role in embryonic development, such as embryonic morphogenesis, brain, eye, and heart development and growth and placenta development, were identified among the HD datasets.

The GO molecular function terms that overlapped among the datasets include protein kinase activity and binding, immunoglobulin binding and biological functions related to the calcium channel. However, the GO cellular components that widely overlapped among the datasets include those related to the nervous system, such as synaptic membrane function, neuronal cell body and pre-synaptic membrane and post-synapse functions.

Protein toxicity is defined as the pathological alternations that ensue from the accumulation, mis-localization and aggregation of disease-specific proteins. Most neurodegenerative diseases have protein toxicity as part of their pathogenic mechanisms. However, the details still remain unclear [22]. Although mutant HTT (mHTT) is the contributing factor to HD pathogenesis, little is known regarding the mechanism by which cytotoxic mHTT is removed from neurons [23]. mHTT is a poor substrate of the known proteolytic pathways of the ubiquitin-proteasome system (UBS), chaperone-mediated autophagy (CMA) and macroautophagy [23]. Studies have shown that mHTT acts an inhibitor of proteolytic mechanisms, often during the protein turnover process [23].

mHTT inclusions in the brain of HD mice and patients were identified to be enriched in the components of the UPS such as ubiquitin and ubiquitinated HTT, as mHTT is initially tagged with ubiquitin but is a relatively poor substrate for the proteasome [23]. Accumulation of mHTT inclusions is not a consequence of proteasomal inhibition, but rather a result from the gross failure of protein quality control systems in association with molecular chaperones [23]

WT-HTT can be degraded by CMA, during which Hsc70 recognizes two KFERQ-like motifs: KDRVN at residues 99–103 and NEIKV at residues 248–252. mHTT is also recognized by Hsc70 for CMA degradation [23]. However, the polyQ expansion of the mHTT delays the delivery of mHTT across the lysosomal membrane, as mHTT has a higher affinity for Hsc70 and LAMP-2A. Failure to promptly deliver the initially targeted mHTT to the lysosome results in a traffic jam in CMA-dependent autophagic degradation, resulting in a secondary side effect of proteostasis. Failure to degrade mHTT results in the accumulation of perinuclear cytoplasmic aggregates and intranuclear inclusions in the neurons of HD patients. The proteolytic pathways in neurons may serve as a target for potential therapeutic strategies to efficiently remove cytotoxic proteins from degenerating neurons.

Oxidative stress is defined as the imbalance of redox homeostasis due to the abnormal increase in free radicals within the cells; free radicals are highly reactive species that result in oxidative damage to DNA, proteins and lipids [24]. Under normal conditions, reactive oxygen species (ROS) also act as signalling molecules, which are involved in growth factor signalling, the activation of immune response, apoptotic pathways, transcriptional processes and several additional biological pathways [25]. The antioxidant defence system regulates free radical production in order to restore redox homeostasis. Antioxidant scavengers include superoxide dismutase (SOD), catalase (CAT), and the thioredoxin (Trx) and glutathione (GPX, GR and GST) systems [24]. Oxidative stress has been observed in HD. A previous study identified oxidative damage in the plasma, post mortem brain tissues, cerebrospinal fluid and lymphoblasts of HD patients. Markers of oxidative stress such as heme oxygenase, 3-nitrotyrosine and MDA were increased in the HD striatum and cortex compared to age-matched control brain tissue [26].

A study by Sánchez-López et al. [27] investigated oxidative stress and inflammation biomarkers in the blood of HD patients. Blood samples were collected from 13 HD patients and 10 age-gender matched controls; the following markers were measured: C-reactive proteins, myeloperoxidase (MPO)/white blood cell (WBC) ratio, interleukin-6 (IL-6), thioredoxin reductase-1 (TrRd-1), thioredoxin-1 (Trx-1), total nitrites (NOx), nitric oxide synthase (NOS) and nitrotyrosine. The results demonstrated that HD is associated with a decrease in TrRd-1 and Trx-1 levels in plasma and erythrocytes and an increase in the MPO/WBC ratio. A positive correlation was observed between global oxidative stress (GOS) and MPO/WBC. However, there were no changes in NOS and Nox levels with respect to controls. Therefore, oxidative damage may be linked to the inflammatory response in HD through a peripheral immune response [27].

Neuroinflammation is another mechanism contributing to HD. Neuronal death can activate inflammatory mechanisms resulting to a vicious cycle of inflammation and neurodegeneration, which contributes to more neuronal damage [28]. Studies have shown that mHTT influences astrocytes and microglia function by inducing the release of inflammatory cytokines including Interleukin-6 (IL-6) [28]. In addition, mHTT positively regulates the NF-kB signalling pathway, inducing the release of inflammatory cytokines and chemokines such as IL-6 and IL-8, respectively [28]. The cytokine profile of HD patients varies from controls, with an increase in IL-4, IL-10 and TNF-α with disease progression [28]. Therefore, there is a vital need for more in-depth research of the sources of free radicals and their targets. For instance, there is evidence that oxidative stress produced by neurons can switch on inflammation by inducing the activation of astrocytes and microglia. In turn, activated brain immune cells produce ROS, pushing forward this endless cycle. However, intervening to stop the signalling pathways that result in the activation of these events may possibly prevent oxidative stress in neurons.

The HTT protein is ubiquitously expressed throughout the body, with the highest level expressed in the brain and testes. In the brain, HTT is found in all neurons [24]. HTT plays a vital role in neuronal survival, stability and function as indicated by the GO cellular component enrichment analysis (Figure 3d and Figure 9d) [29,30]. Over the past years, the role of HTT during embryonic development has been investigated. Our study also identified numerous GO biological processes involved in embryonic morphogenesis and brain, eye, heart and roof of mouth development (Figure 3b and Figure 6b) at the pre-symptomatic HD stage (Figure 3 and Figure 6), along with regulation of neurogenesis, placenta development (Figure 4b), gland development and appendage development (Figure 5b) for symptomatic HD stages. There are studies that support the theory that abnormal brain development may play a role in HD pathology [30]. WT-HTT is essential for brain development; embryos of HTT knock-out mice demonstrated serious abnormalities in CNS development, resulting in their death shortly after birth [30]. Therefore, given the importance of HTT in development, a partial loss of function may contribute to abnormal neural development. Although HD pathology indicates that mHTT results in a gain-of-function that leads to neural damage, there is evidence that, in addition to these mechanisms, the loss of function of WT-HTT also plays a part in the disease mechanisms [30]. A study by Nguygen et al. [31] demonstrated that HTT is essential for neural induction, specification of neural progenitor cell types and the successive elaboration of neural lineage species [31]. Furthermore, mHTT has been shown to result in impairments at multiple stages of striatal development, supporting the idea that selective vulnerability of striatal neurons may have a developmental pathoetiology [31]. How exactly embryonic development and, more specifically, brain development are affected in HD individuals during their early stages of development remains unclear. However, despite these areas of vagueness, considerable progress has been made, in particular regarding the development of new treatments and possible prevention of HD in the context of gene therapy approaches during the early stages of embryonic development.

The complement system is tightly regulated and an important component of the innate immune system, which recognizes and eliminates invading pathogens, dead or modified self-cells such as cellular debris and apoptotic cells. It also plays a role in immune surveillance, maintenance of cellular integrity, tissue homeostasis and neuroprotection [32] (Figure 3a and Figure 4a) during the pre-symptomatic and symptomatic HD stages. In addition, the GO molecular function terms of immunoglobulin receptor binding (Figure 3b and Figure 5b) and cytokine binding (Figure 7b), which are immune system functions, are also involved in the HD stages. Activation of the complement cascade occurs via three major pathways: (i) the classical pathway, (ii) the mannose-binding lectin pathway and (iii) the alternative pathway. Each pathway is activated by a different stimulus type but produces the same effector molecules. The production of C3 triggers downstream effectors of the activated complement cascade, which is a common basic feature of the three complement pathways [32]. There is growing evidence implicating the role of the complement system in HD. Studies have shown that aberrant complement up-regulation and microglial activation have been observed in both pre-clinical mouse models and human brains [32]. The proteins of C1q, C4, C3, iC3b and C9 of the classical complement pathway were identified to be up-regulated in the striatum of human HD post-mortem brains. In addition, the proteins were localized to neurons, myelin and astrocytes [32].

A previous study by Liddelow et al. [33] observed the up-regulation of the C3 mRNA transcript in the prefrontal cortex and abnormal C3 immunoreactivity on reactive astrocytes in the caudate nucleus of human HD post-mortem brain tissues [33]. Striatal C3, C9 and C5aR proteins were identified to be up-regulated in the 3-nitropropionic acid (3-NP) HD rat model. Furthermore, increased serum C3 level has also been observed in HD patients, suggesting the role and involvement of the complement alternation in HD [33].

Proteomics analysis between HD patients and healthy controls revealed increased C7, C9 and clusterin expression levels in plasma and CSF [33]. At the same time, the mRNA transcripts of classical complement initiators that include C1r, C4 and C3, the receptors of C3aR and C5aR and the regulators of C1 inhibitors, clusterin, MCP, DAP and CD59 were observed to be up-regulated in human HD brains compared to healthy controls [33].

The investigation of additional complement components and proteolytic fragments may aid in illuminating the relationship between the complement cascade activation and HD pathogenesis. Further studies, are needed to evaluate if inhibition of the complement cascade could serve as a potential therapeutic target for HD.

Clustering was performed to identify the closely connected gene modules within the PPI network of the 128 common gene targets from the public available databases of CTD, Malacards and Disease Database (Figure 10). Some of the genes identified in cluster 1 (Table S18), such as *CASP3*, *CASP8*, *CASP1* and *FoxO3*, play a role in apoptosis. The antioxidant gene of *SOD1*, which neutralizes free superoxide radicals in the body, and genes involved in fatty acid storage and glucose metabolism, such as *PPARG* and *IGF-1* [34], were also identified.

Some cluster 2 (Table S19) genes obtained, such as *PPARGC1A*, were identified to have a significant role in energy metabolism; HD patients are known for their decrease in weight although they consume foods with higher caloric intake. The *BDNF* gene is vital for neuronal survival and differentiation of neuronal cells during development [34]. *NFKB1* is a transcriptional regulator, activated by several intra- and extra-cellular stimuli such as cytokines, oxidant free radicals, bacterial or viral products and irradiation. Inappropriate activation of NFKB has been associated with a number of inflammatory diseases while persistent inhibition of NFKB results inappropriate immune cell development and delayed cell growth [34].

Cluster 3 genes (Table S20) included genes such as *ACHE* that encode for the cholinesterase enzyme that catalyses the breakdown of acetylcholine in the post-synaptic neuron [34]. *GRIK2* encodes for glutamate receptors, which are predominantly excitatory neurotransmitter receptors, and it is activated in various neurophysiological processes [34].

Enrichment analysis graphs of each MCODE cluster using KEGG identified mainly neurodegenerative (HD, AD, PD), signalling pathways (cAMP, Rap1 and calcium signalling pathways) and synaptic pathways (glutamatergic and cholinergic synapse). Furthermore, the GO biological process, molecular function and cellular components identified a number of mechanisms and cell components involved in the CNS that include pre-synaptic and regulation of post-synaptic membrane potential, synaptic membrane and vesicles.

In HD, the cholinergic striatal interneurons are thought to be spared. However, recent studies have observed that neuronal dysfunction with no cell death may also contribute to the early- and mid-stages of HD [35]. A hypothesis suggests that dysfunctional acetylcholine (Ach) neurons may release in the brain, contribute to learning, memory and behavioral impairment [36]. The study by Smith et al. [35] investigated that cholinergic transmission is affected in the R6/1 HD transgenic mouse model and in the tissues from HD patients [35]. The stereological analysis performed showed no loss of cholinergic neurons in the striatum or septum in R6/1 mice, in contrast to the mRNA and protein levels for the vesicular acetylcholine transporter (VAChT) and choline acetyltransferase (ChAT), which are reduced in the striatum and cortex, and acetylcholine esterase activity, which was found to be lower in the striatum of R6/1 mice at a young age [35].

Reduced VAChT was also seen in patient samples, but it was restricted to the striatum. Furthermore, the localization and expression of REST/NRSF, which is a transcriptional regulator for the ChAT and VAChT genes, was not altered in cholinergic neurons [35]. The above-mentioned study observed that the R6/1 mice display severe deficits in learning and memory [35]. Therefore, the cholinergic system become dysfunctional in R6/1 transgenic mouse models and HD patients. The preceding may provide a foundation for testing pro-cholinergic drugs in HD and may benefit HD patients.

The topological network parameters selected via CytoHubba revealed the genes of *CASP3*, *MAP2*, *GRIN2A, FoxO3*, *ITPR1*, *PPARGC1A* and *CREBBP* to be highly ranked based on the topological network measures of MCC, degree, closeness, betweenness, eccentricity and clustering coefficient that were applied (Table 5 and Figure 13).

The PPI network (Figure 12) of the 18 HD-related hub genes showed that Caspase-3 (*CASP3)* interacts with various proteins such as *FoxO3*,which also has a role in apoptosis [37], but also with proteins such as peroxisome proliferator activated receptor gamma co-activator 1-alpha (*PPARGC1A*), which regulates the gene involved in energy metabolism [38], and microtubule associated protein 2 (*MAP2*). Members of the MAP family are known for their role as microtubule stabilizing activity and their roles as regulators of microtubule networks in the dendrites and axons of neurons [39].

CASP3 is a key mediator of apoptosis; CASP3 is a frequently activated death protease, which catalyses specific cleavage of numerous cellular proteins [40]. A study by Sanchez and Friedlander [41] demonstrated in a HD transgenic mouse model that CASP1 and CASP3 are both transcriptionally activated and up-regulated. Findings have suggested that CASP1 is activated in the brains of HD patients [41]. Caspase activations results in the proteolytic cleavage of key cellular targets, including HTT, that leads to cellular dysfunction and death which correlates with HD disease progression [41]. Furthermore, caspase inhibition in HD transgenic mice resulted in an observed delayed onset and progression of symptoms and prolonged survival. Therefore, caspase inhibition may act as a future potential therapeutic strategy, and further research is required to investigate and evaluate this in patients suffering with HD [41].

The Forkhead box O (*FoxO*), and more specifically FoxO3, is a transcription factor that has the ability to be inhibited and translocated out of the nucleus on phosphorylated proteins such as Akt/PKB and PI3K signalling pathways [42]. Furthermore, FoxO3 functions as a trigger for apoptosis via the up-regulation of Bim and PUMA, which are vital for cell death, or the down-regulation of anti-apoptotic proteins such as FLIP [37]. FoxO transcription factors can exert both damaging and protective effects on neurodegenerative diseases.

In HD, FoxOs have a significant protective role by clearing mHTT during the early HD stages [43]. Increased proteasome and autophagy activations are an essential mechanism for mHTT clearance. Furthermore, increased FoxO levels are associated with enhanced proteasomal activity in HD induced-pluripotent stem cells (iPSCs) [43].

FoxO3 activity was observed to be increased in both mouse striatal Hdh neurons and in the cortex of R6/2 mice, where progressive symptoms of HD were observed [43]. A comparable increase in FoxO3 activity was also observed in post-mortem caudate tissues from HD patients in different HD stages. Subsequent studies confirmed that an increase in FoxO observed with HD may represent a neuroprotective mechanism [43].

An increased level of FoxO3 mRNA transcripts were detected in HD patients due to an over-activated positive auto-feedback loop, in which FoxO3 binds to the FoxO3a promoter and regulates its own transcription [43,44] The overactive positive feedback loop of FoxO3 is suggested to be an early protective mechanism for neurons in HD patients. The Wnt receptor Ryk has a vital role in neuronal differentiation, and it increases cell death by inhibiting FoxO3 activity in mHTT neurons, suggesting that suppressed FoxO3 activity attenuates the function of neurons during HD [43]. Furthermore, XBP1 has been implicated in HD pathology as a suppressor of FoxO1 expression; XBP1 deficiency and ectopic expression of FoxO1 may protect against HD by inducing autophagy [43].

A study by Sadagurski et al. [45] reported an increased nuclear accumulation of FoxO1 and the expression of *SOD2* and *PPARGC1A*, FoxO1-dependent genes, in the brains of R6/2 diabetic mice with increased insulin receptor substrate 2 levels; these mice had slower progression of HD symptoms [45].

The miRNA-gene network (Figure 14) identified the eleven miRNAs of mir-19a-3p, mir-34b-3p, mir-128-5p, mir-196a-5p, mir-34a-5p, mir-338-3p, mir-23a-3p and mir-214-3p based on HMDD (Figure S5). The miRNA identified in the network have been identified to either target the HTT gene (mir-214-3p, mir-34a-5p), have the potential to be used as a diagnostic biomarker (mir-128-5p, mir-34b-3p, mir-128-5p, mir-338-3p and mir-23a-3p) or as a potential therapeutic target (mir-128-5p). Furthermore, the preceding miRNAs interacted with some HD-related genes that were shared between the miRNA gene targets, HD DEGs and the hub genes. A total of eight genes were identified: *ITPR1*, *CASP3*, *GRIN2A*, *FoxO3*, *TGM2*, *CREBBP*, *MTHFR* and *PPARGC1A*; the aforementioned genes play a role in apoptosis, embryonic development, interactions with HTT, energy metabolism and channels that controls the flow of positively charged calcium ions within cells. The latter play a role in maintaining calcium homeostasis, mitochondrial functions or have a role in signalling pathways [34].

To the best of our knowledge, this is the first study integrating data from various public databases to identify common pathways and mechanisms of HD in the pre-symptomatic and symptomatic HD stages and potential genes and miRNAs that may be targeted as novel pharmacological targets for the treatment of pre-symptomatic and symptomatic HD stages. Gene expression studies can help us to better understand the specific pathobiology in HD. This can pave the way for better drug development and diagnostic biomarkers. Our study is not without its limitations, which include (i) the use of three HD datasets as most of the datasets were in mice and (ii) a lack of experimental validation of our findings in vivo and in vitro. Future work can focus on (i) the microbiota bioactivity in relation to oxidative stress and its effect on the inflammatory process and dysbiosis in pre-symptomatic and symptomatic HD patients, (ii) experimental validation of findings, and (iii) a combination of nutritional systems biology which focuses on nutritional intake and measures these consequences as accurate omics signals to identify macro and micronutrient deficiency that can lead to neurodegenerative disease and how a combination of nutritional information and omics data may be a future pre-therapeutic possibility for HD. In conclusion, our work revealed that various biological pathways seem to be involved in HD during the different HD stages such as pre-symptomatic HD or symptomatic HD. There is growing evidence that supports the hypothesis that embryonic development, and more specifically brain development, is affected in HD. Furthermore, pathways and genes involved in apoptosis, synaptic transmission and the immune system were also identified. In addition, we identified novel gene candidates and miRNAs that could be further investigated and used as potential biomarkers or as therapeutic targets. This may offer some clues for the molecular mechanisms, pathways and cellular components underlying HD. However, the results of the study need to be further validated in vitro and in vivo, which will be a critical direction for future research in the field of pharmacological development and to determine at which stage intervention should start.

## 4. Materials and Methods

**Figure 15:**
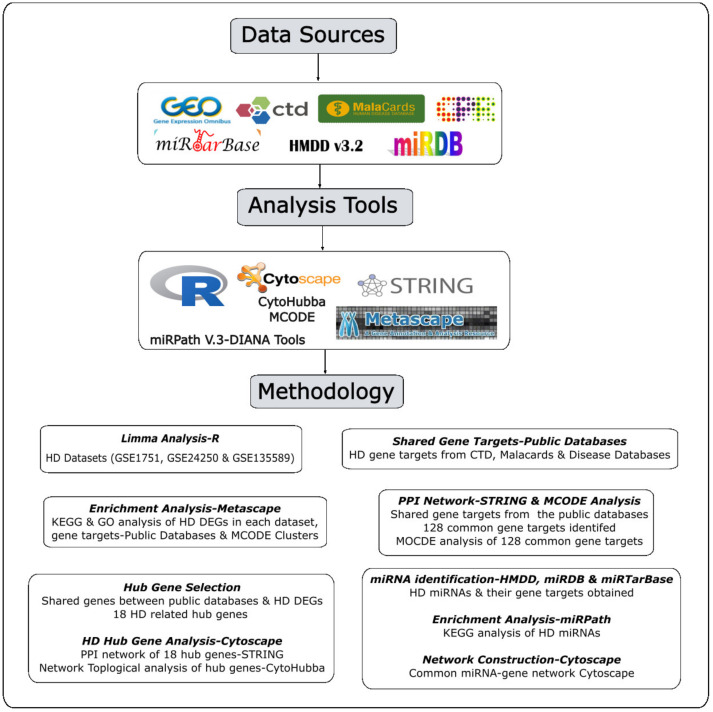
Workflow methodology applied in the study.

The workflow applied in this study is shown below as Figure 15.

### 4.1. Data Sources

The Gene Expression Omnibus (GEO) of the National Center for Biotechnology Information (NCBI) database [46] was extensively searched for datasets that meet the following criteria: (i) consisting of two HD stages, or one HD stage versus controls and ii) the biological fluid being blood. Three transcriptomics datasets with the accession numbers GSE1751 [8], GSE24250 [9] and GSE135589 [10], respectively, were found fitting these criteria. Details on the collection of peripheral tissue samples for each dataset can be found in detail in their respective papers [8].

In addition, the public databases of the Comparative Toxicogenomic Database (https://ctdbase.org/ accessed on 19 December 2022) [47], Malacards (https://www.malacards.org/ accessed on 20 December 2022) [48] and (iii) Disease database (https://diseases.jensenlab.org/Search accessed on 21 December 2022) [49] were used to obtain HD-related genes.

### 4.2. Data Processing and Differenetial Gene Expression Identification

The linear models for microarray data (Limma) is an R package used for the analysis of gene expression data obtained from microarray and RNA-Seq experiments that allows for the identification of differential expressed genes (DEGs) between different disease stages vs controls, case vs controls or treated vs non-treated individuals [50]. To identify the DEGs between controls and HD samples from the 3 datasets individually, Limma was used [50]. The datasets were normalized using a log_2_-transformation and an adjusted *p*-value of <0.05 indicated statistical significance.

A total of 400 DEGs (top 200 over- and top 200 under-expressed genes with adjusted *p*-values < 0.05) were selected for each HD dataset. In the case of the GSE135589 dataset, there are 400 DEGs for Year 1 and 400 DEGs for Year 3. To avoid noise within our networks and to acquire meaningful information from each of the networks, a further cut-off threshold was applied to the final edgelist for all three datasets. The weights from the final edgelist were converted to a log function (log(weight)); therefore, genes and their weights of 1 and above were used as inputs in Cytoscape for the construction of gene co-expression networks.

### 4.3. Enrichment Analysis of DEGs

Metascape is a web-based tool that allows for comprehensive gene list annotation and enrichment analysis for an assortment of different organisms; it combines enrichment and interactome analysis, gene annotation and protein–protein interactions [12].

Metascape is a meta-analysis tool that provides a better understanding of either common or unique pathways or protein–protein interaction networks in a disease of interest [12]. Furthermore, terms with a *p*-value of <0.05, a minimum count of 3 and an enrichment factor of >1.5 (the ratio between the observed count and the counts expected by chance) are grouped into clusters based on their membership similarity.

*P*-values are calculated based on the accumulative hypergeometric distribution and q-values are based on the Benjamini-Hocherg method for multiple testing. Kappa Scores are used for the similarity metrics to perform hierarchical clustering on the enriched terms obtained, and sub-trees with a similarity of >0.3 are clustered together; only the most statistically significant terms are chosen to be represented within the cluster [12].

In order to analyse the biological pathways and functions involved in HD, the KEGG and GO libraries were used to perform enrichment analysis using Metascape [12]. The names of the top 200 over- and under-expressed genes identified for each dataset individually were used as inputs into Metascape and the organism *Homo Sapiens* was selected. The KEGG and GO-Biological Processes libraries were selected for enrichment analysis for the identification of biological pathways and processes; the *p*-value cut-off was set to <0.05.

### 4.4. Shared Gene Targets from Public Databases

The three publicly available databases of the Comparative Toxicogenomic Database (https://ctdbase.org/ accessed on 19 December 2022) [47], Malacards (https://www.malacards.org/ accessed on 20 December 2022) [48] and Disease (https://diseases.jensenlab.org/Search accessed on 21 December 2022) [49] were used to obtain the most common HD-related genes. The intersection of the Venn (https://bioinfogp.cnb.csic.es/tools/venny/ accessed on 27 December 2022) diagram was used to identify the number of shared gene targets in HD among the three databases.

### 4.5. PPI Network of Shared Gene Targets from Public Databases

The 128 common gene targets obtained from the CTD [47], Malacards [48] and Disease databases [49] were then used in STRING [13] to construct a PPI network with a combined score greater than 0.4. The PPI network was then imported to Cytoscape, and the MCODE plug-in in Cytoscape [11] was used to identify the closely connected gene modules within the PPI network. The MCODE parameters applied to the PPI network were: (i) Find and report clusters within the whole network: Here, clusters will be found and reported in the entire network; (ii) A degree-cut off of 2 was applied: The degree-cut off controls the minimum degree (number of connections) necessary in order for a node to be scored; (iii) Hair-cut clustering finding was applied: Here, once a cluster had been found, some nodes which may have satisfied the degree cut-off parameter during scoring may only be connected to the cluster by 1 edge. Using this parameter removes all singly connected nodes from clusters; (iv) K-core filter of 2: This filters out clusters that do not contain a maximally interconnected sub-cluster of at least *k* degrees; (v) Maxdepth of 100: This parameter limits the distance from the seed node which MCODE can search for cluster members [14].

Cluster rank is based on the clusters’ computed score and used to identify the clusters within each result. Therefore, cluster 1 is the highest-ranked cluster.

### 4.6. Hub Gene Selection

Genes that interconnect with multiple other genes are defined as hub genes and usually play a vital role in a biological mechanisms and processes [51]. To identify the hub genes present, the common gene targets obtained through the CTD [47], Malacards [48] and Disease [49] and the DEGs identified for each HD-related dataset were used as input. The intersection of the Venn (https://bioinfogp.cnb.csic.es/tools/venny/ accessed on 27 December 2022) diagram was used to identify the hub genes shared between the three public databases and DEGs identified using Limma for each HD dataset.

Biological networks include PPI, metabolites, gene regulations, miRNA-genes, cellular networks and signal transduction pathways. Topological network parameters have the ability to measure nodes within the network to infer their importance within the biological network helping to identify central biological entities within the network of interest [15]. The 18 HD-related hub genes were exported from STRING with a medium confidence of 0.400 into Cytoscape [11,13]. The CytoHubba [15] plug-in in Cytoscape [11] ranks nodes within the network by their network features; it is able to analyse a network using eleven scoring methods, which include (i) maximum neighbourhood component (MNC), (ii) density of maximum neighbourhood component (DMNC), iii) maximal clique centrality (MCC), (iv) degree, (v) closeness, (vi) eccentricity, (vii) radiality, (viii) bottleneck, (ix) stress, (x) betweenness and (xi) edge percolated component. For further detail regarding each network parameter, please refer to paper [15].

The parameters used for the CytoHubba analysis of the hub gene network were the top 10 nodes ranked by (i) MCC, (ii) degree, (iii) closeness, (iv) betweenness, (v) eccentricity and (vi) clustering coefficient [15]. Red- to yellow-coloured nodes represent the highest to lowest ranking of nodes based on the topological approach used, while the edges represent the interactions between the genes within the network.

### 4.7. Identifiying the Common miRNAs

microRNAs (miRNAs) are a class of small non-coding RNAs which have vital roles in regulating gene expression [52]. miRNAs can regulate transcription and activate translation and can modulate gene expression by promoting or inhibiting mRNA degradation and translation [52]. Furthermore, miRNAs can be secreted into extracellular fluids and transported to target cells via vesicles such as exosomes [52].

Therefore, we investigated whether some miRNAs share common regulatory mechanisms or biological processes in HD. The HD-associated miRNAs were obtained from the Human miRNA Disease Database (HMDD) (https://www.cuilab.cn/hmdd accessed on 9 January 2023) [53,54,55]. HMDD is an up-to-date detailed and comprehensive annotation database of the human miRNA–disease associations. The database includes miRNA–disease association data such as genetic, epigenetic and circulating miRNAs evidence and miRNA–target interactions [53,54,55]. In addition, the miRDB online database [16,17] was used to identify if the HD-related miRNAs have a 5-prime arm (5p) of the hairpin loop or a 3-prime end (3p), as the miRNAs identified for HD were too ambiguous to perform enrichment analysis using mirPath v.3 [18]. Furthermore, KEGG pathways enrichment analysis was performed on the HD-related miRNAs using the online software mirPath v.3 [18] from DIANA tools [19].

### 4.8. Common miRNA-Gene Network Construction

The gene-target information for each of the HD-associated miRNAs was identified using the miRTarBase (https://mirtarbase.cuhk.edu.cn/ accessed on 12 January 2023), an experimentally validated miRNA–target interaction database [20]. The HD miRNA-gene network was constructed by taking the intersection of the Venn diagram (https://bioinfogp.cnb.csic.es/tools/venny/ accessed on 13 January 2023) for the miRNA gene targets obtained from miRTarbase, HD DEGs obtained for all HD datasets and the 18 hub genes identified. The target genes of the common miRNAs in HD, the shared genes in HD and the hub genes were used to construct the miRNA-gene network using Cytoscape [11,56].

## Figures and Tables

**Table 1:** Detailed information of the three transcriptomics datasets of HD analysed in this study.

Accession ID	Platform	Platform Type	Sample Size (Case/Control)	Sample Type
GSE1751	GPL96	Affymetrix Human Genome U133A Array	31 (12 symptomatic, 5 pre-symptomatic and/14 controls)	Peripheral Blood
GSE24250	GPL96	Affymetrix Human Genome U133A Array	14 (8 HD patients/6 controls)	Whole Blood
GSE135589	GPL570	Affymetrix Human Genome U133 Plus 2.0 Array	* 178 (Year 1: 18 Stage 1 HD, 19 Stage 2 HD/24 controls)	Peripheral Blood
			(Year 3: 16 Stage 1 HD, 16 Stage 2 HD/20 controls)	

* Consists of 17 HD-mutation positive pre-symptomatic far from age of predicated onset (Pre-A) and 18 HD-mutation positive pre-symptomatic near to age of predicated onset (Pre-B); groups were not included in the analysis as no significant DEGs were obtained.

**Table 2:** Shared co-expressed genes in merged networks for controls versus pre-symptomatic and controls versus symptomatic HD datasets (GSE1751).

Shared Co-Expressed Genes in Merged Pre-Symptomatic and Symptomatic Networks		
*MYBL1*	*LRRC40*	*ATF2*
*MATN1*	*CNTN6*	*DCHS1*
*DMXL1*	*SMAECA5*	*HYAL1*
*IRAK3*	*DENND2A*	*MAP4K3*
*XYLB*	*ALG13*	*RAD51*
*TRMT13*	*PLXNB2*	*PMAJP1*
*OXR1*	*RBM25*	*PHTF2*
*NBN*	*NUPL1*	*SPAST*
*ZNF107*	*RAB2A*	*ZNF267*
*BAZ2B*	*LTN1*	*ZNF83*
*TMED7/TMED7-TICAM2*	*APPL1*	*PSMC6*
*ZNF518A*	*ZDHHC17*	*CD46*
*MOB1A*	*HSDL2*	*DEK*
*STAM2*	*PNISR*	*DCAKD*
*ITGA4*	*PPP1R12A*	*GOLGA8N*
*HNMT*	*PICALM*	*PIKFYVE*
*NDUFA4*	*RPS7*	*CHMP5*
*ASF1A*	*PPP1CB*	*BMI1/COMMD3-BMI1*

**Table 3:** Shared co-expressed genes in merged networks for controls versus Stage 1 (Year 1) and controls versus Stage 2 (Year 3) datasets (GSE135589).

Shared Co-Expressed Genes in Merged Stage 1 (Year 1) and Stage 2 (Year 3) Networks		
*N4BP2L2*	*SFT2D1*	*TTLL5*
*CEBPZOS*	*BC039537/RP11-30L15.6*	*NFIB*
*BCAS4*	*TBC1D12*	*MGC57346*
*TAF1A-AS1*	*GUCY1A3*	*GYPA*
*GNG11*	*THBS1*	*HTR2A*
*ZNRD1-AS1*	*GUCY1B3*	*BCL2L1*
*PBRM1*	*TBC1D22B*	*TMCC2*
*HPS1*	*ELL2*	*ABCC4*
*CTSE*	*KEL*	*SLC6A10P/SLC6A10PB/* *SLC6A8*
*SLC6A8*	*LOC100996902*	*ABCC13*
*SOX6*	*HBM*	*C7orf73/LOC101930655*
*MARCH8*	*EPB41*	*SESN3*
*RNF182*	*GABPB1-AS1*	*ANK1*
*LOC101927507*	*ARG1*	*TRIM58*
*FAXDC2*	*OSBP2*	*SLC2A1*
*TNS1*	*IGLC1/IGLV9-49*	*SMIM24*
*FKBP1B*	*HBD*	*TGM2*
*PROS1*	*PAQR9*	*ASPM*
*SPARC*	*ELMOD2*	*SDHD*
*OTUD6B-AS1*	*FAR2*	*MS4A3*
*CDH17*	*FSBP/RAD54B*	*PHOSPHO2*
*DEFA4*	*EPHA4*	*ADAM12*
*GABPB1-AS1*	*RNF182*	*SESN3*

**Table 4:** The HD-related hub genes.

Gene Name		
*CASP3*	*MAP2*	*PPARGC1A*
*CREBBP*	*GRIN2A*	*ITPR1*
*FOXO3*	*IFT57*	*FMR1*
*MTHFR*	*TGM2*	*GRIK2*
*IDUA*	*FOLH1*	*TCERG1*
*PCSK2*	*PCM1*	*ATXN3*

**Table 5:** The ranking hub genes identified by Cytohubba for the six network scoring measures of the 18 HD-related hub PPI network.

Gene Name	MCC	Degree	Closeness	Betweenness	Eccentricity	Clustering Coefficient
*CASP3*	6	6	8.6	94	0.24	0.06
*MAP2*	2	2	6.5	54	0.24	0
*GRIN2A*	3	3	5.9	42	0.18	0
*FoxO3*	7	5	7.8	37	0.18	0.3
*ITPR1*	2	2	6.1	7	0.18	0
*PPARGC1A*	4	3	6.8	7	0.18	0.66
*ATXN3*	3	3	6.15	4	0.14	0.33
*CREBBP*	4	3	6.15	1	0.14	0.66
*IFT57*	1	1	5.3	0	0.18	0
*TGM2*	1	1	5.3	0	0.18	0
*TCERG1*	1	1	4.9	0	0.14	0
*GRIK2A*	1	1	4.1	0	0.14	0
*FMR1*	1	1	4.1	0	0.14	0
*FOLH1*	1	1	1	0	0.11	0
*MTHFR*	1	1	1	0	0.11	0
*IDUA*	0	0	0	0	0	0
*PCSK2*	0	0	0	0	0	0
*PCM1*	0	0	0	0	0	0

## Data Availability

The publicly available datasets of GSE1751, GSE24250 and GSE135589 used in the following study were obtained from the Gene Expression Omnibus (GEO) (https://www.ncbi.nlm.nih.gov/geo/ accessed on 30 November 2022). All data used for the following study was obtained from public databases.

## Supplementary Materials

The following supporting information can be downloaded at: https://www.mdpi.com/article/10.3390/ijms24054873/s1.
