# Supplementary material for: Integrated Bioinformatics Analysis of Shared Genes, miRNA, Biological Pathways and Their Potential Role as Therapeutic Targets in Huntington’s Disease Stages
Source: Int J Mol Sci. 2023 Mar 2;24(5):4873. doi: 10.3390/ijms24054873 (PMC10003639; doi:10.3390/ijms24054873)
Supplement: Supplementary file 1 [file ijms-24-04873-s001.zip › ijms-2239296-supplementary.pdf]

Table S1. Over-expressed genes in controls versus pre-symptomatic HD.

Over-expressed genes			
OXR1	STAG2	RUFY3	CHMP5
ITGA1	BAG5	DNAJB14	ZNF292
GREB1	QKI	PIKFYVE	SUSD5
USP34	LAMB1	RBM25	MAP4K3
CD46	C8orf44-SGK3 /// SGK3	SLC30A10	LOC389906
NABP1	AHR	KLHDC10	MCFD2
PLEKHS1	DMXL1	DNAJB9	ZNF83
C5orf28	NDUFAF7	CCND1	NCOA2
STAM2	LAMA2	PKN2	DYRK2
PSMC6	NBN	RRP15	TRMT11
DLG1	GLCE	ZNF675	LIN7C
GNAL	BAZ2B	ITSN1	PHTF2
PLEKHB2	GRTP1	PCM1	PIK3R4
SPAST	SLC30A1	YEATS4	DNAJC15
LRRC40	SH3GL2	SNAPC1	WDYHV1
NUPL1	NBPF10 /// NBPF14 /// NBPF26 /// NBPF9	RNF41	IRAK3
ZNF267	CD59	SMARCA1	ZNF518A
DICER1	STAT1	BBS10	IFT57
PLOD2	GOLT1B	COX7C	TARDBPP1
PMAIP1	BLZF1	MOB1A	TIA1
FASTKD3	USP14	MIR22 /// MIR22HG	ITGA4
KRR1	SMARCA5	SNX6	PHIP
ZNF107	SATB2	MYBL1	PLEKHA1
FRMD4A	GNL3L	SI	ATF2
LAMA4	NRIP1	BNC1	WWTR1
BBS7	NR5A2	ADCY8	RPS7
PCNXL4	Y16709	FKBP15	GRIN2A
URI1	TTC3 /// TTC3P1	DDX17	LOC101060363 /// PPIA
CADM1	PPP1CB	ASF1A	DEK
PNISR	TMED7 /// TMED7-TICAM2	TLK1	AIMP1
RNF128	GATAD1	CD58	ITPR1
IMPACT	SLC12A1	ALG13	SLC25A21
IREB2	GOLGA8N	TMEM5	HSDL2
SYNJ1	IFIH1	CPM	CRBN
HAUS6	MPPE1	RCN2	EIF5
PRKCI	NIF3L1	TNPO1	TRMT13
ZDHHC17	PLGLB1 /// PLGLB2	GATA3	AGBL3
NEFL	NFATC4	EBAG9	CDC37L1
GOLGA6L4 /// GOLGA6L5P /// GOLGA6L9 /// LOC102724093	NCAPG	XYLB	IGLVIVOR22-1 /// IGLVIVOR22-1
CAPZA1	CCHCR1	CCDC81	UGCG
RBM15	RAB2A	LTN1	CLGN
BMI1 /// COMMD3-BMI1	PHLDA2	PICALM	HBZ
ORC5	PDE3B	RAD52	CRIM1 /// LOC101929500
FERMT2	LRRC14	FAM115A /// LOC100294033	MALT1
NDUFA4	MARCH1	FASTKD2	GRAMD1C

ZNHIT6	GUSBP3 /// GUSBP9	AGTR2	THAP9
PPP1R12A	INHBA	EXTL1	MED6
ATP8A1	IL18	CREBZF	PTPRN2
HNMT	NUS1P3	CAPN7	APPL1
SLC22A3	TRIM24	RAB3GAP2	
**Under-expressed genes**			
KLHL41	MIR1236 /// NELFE	CACNA1I	PPY
TUSC3	IKBKAP	ARSE	PRAMEF12
RPS4XP3 /// RPS4XP3	EDA	TAC1	FEZF2
GPR4	AVPR1A	TFAM	LOC100506699
MTFR1	IGHG1 /// LOC101930405	JMJD6	NTN1
DENND2A	BTBD18	RAD51	C4orf19
TMEM63A	HOXC8	DOHH	HPS4
NRG2	LUZP1	GINS4	DFNA5
SRPR	TPSD1	C14orf105	PP13
SH2B1	DLGAP4	HLA-DRB4	AQP5
KCNQ1DN	MTHFR	CFB	TPSAB1
DCHS1	NBAS	COL6A1	PRSS50
NF2	PIAS2	SGSM2	PRRG2
NID1	CALML3	SOX12	DLEC1
EPHB2	DGCR5	HARS /// LOC101928623	SENP3
DCAKD	ZDHHC8P1	CRNN	PCSK1N
RND2	CNTN6	LRRC48	NARFL
DDR2	ATP1B4	DOCK6	SKIL
MDM2	CDK16	HPGD	MSLN
UGT1A1 /// UGT1A10 /// UGT1A4 /// UGT1A6 /// UGT1A8 /// UGT1A9	IGFBP7	GRB7	MIR4800 /// MXD4
CCKBR	GNRH2	MAP2K5	MED22
TAF6L	CLTA	RET	ADH6
SIGIRR	FAM215A	HNRNPA1P37 /// HNRNPA1P37	GCNT2
LOC101929910 /// LOC613037 /// NPIPA5 /// NPIPB11 /// NPIPB3 /// NPIPB4 /// NPIPB5 /// NPIPB8	ACADL	NR2F1	HYAL1
KCND2	ATP8A2	PDLIM7	NDRG4
DHRS1	MAFK	E2F2	CD72
IVD	MAP2	OLIG2	MAGEB4
TMEM100	TKTL1	MIP	ESRRG
SYNPO2L	BEX4	LOC102724905	CDK5R1
ARID1A	DHX34	CTNNA2	GTF2F2
TGFB1I1	NAV2	LEPREL1	CNN1
PLXNB2	LAMC1	KMT2A	FBXO40
ABCC2	RPL29P7	OCA2	ORM1
B3GAT1	HDLBP	XAB2	WDR76
MARS /// MIR6758	SHH	AMBRA1	FAM134B
ZNF749	MATN1	ZXDC	YAP1
ACVR1B	LSS	USP222	PLP1

FBXL6	MRPL40	PF4V1	TFR2
SPRR1A	CCDC70	TMEM255A	HIP1
PITX2	RANBP3	KCNB2	FOCAD
TFAP2A	IGHA1 /// IGHG1 /// IGHM /// IGHV3-23 /// IGHV4-31	ERBB4	MGC2889
LEPRE1	TNFRSF12A	SIRT5	ACLY
MRPS34	FUCA1	TAPBPL	MAP7
A2M	TSPAN1	IQCK	ABCA2
KIAA0586	PRMT8	UTP14A	ARMC9
SORT1	MAP1S	DMP1	PRCC
IL12RB2	LOC440434	OTC	ZNF227
CCDC177	TMEM57	ACOT11	UBA6
GRIK2	CAND2	ZSCAN5A	MYL9
IL1RN	TIMM44	SOAT2	

Table S2. Over-expressed genes in controls versus symptomatic HD.

**Over-expressed genes**			
ZNF267	CHMP5	ATF1	PIKFYVE
C12orf29	STAM2	SPAST	ANKRD12
SUB1	UFL1	RPS7	GLS
ZNF107	CLK1	PMAIP1	ZDHHC17
PHTF2	MOB1A	PRPF4B	TMEM168
PSMC6	CHMP2B	SP3	PPP1R12A
LRRC40	ATF2	FAM188A	DMXL1
OSBPL8	RB1	ASF1A	TMED7 /// TMED7-TICAM2
EDEM3	HSPE1-MOB4 /// MOB4	PHIP	ACSL4
HNMT	AHR	C2CD5	MBNL2
EVI2A	SH2D1A	DR1	TRMT13
ATP8A1	RAP2C	PKN2	ARGLU1
BAZ2B	PPIP5K2	COX7B	GOLGA8N
MYBL1	NAB1	NBN	MAN1A1
ABHD3	ALG13	C6orf211	LTN1
ARID4A	MAP4K3	RBM25	ZNF83
PLSCR1	CASP3	HINT1	BMI1 /// COMMD3-BMI1
NEK7	LOC102724200 /// TRAPPC10	PCNP	SMARCA5
PNISR	CEP135	TVP23B	RBM15
KIAA1033	DEK	NDUFA4	SLC25A24
TANK	ZFYVE16	CD46	IRAK3
SEC23A	OXR1	COPS2	TMEM165
SACM1L	MTHFD2	CLEC2B	KLF9
KRIT1	CMPK1	NOL8	TMED2
GK	KIAA1109	SNX10	EID1
COMMD8	ZNF518A	ANKRD49	MICU2
ANP32E	SLC35A1	CCP110	CLDND1
HAT1	ACTG1P4 /// AMY1A /// AMY1B /// AMY1C /// AMY2A /// AMY2B	AIMP1	TWF1
CRK	SLC35A5	CXCL8	RAB2A
RPL9	RNF138	MEF2A	PPIG

NRIP1	CLINT1	MIR1304 /// SNORA1 /// SNORA18 /// SNORA32 /// SNORA40 /// SNORA8 /// SNORD5 /// TAF1D	MIA3
ERAP1	NAMPT	CDC14A	ERGIC2
CDC73	STK3	PICALM	ERBB2IP
IFRD1	ST3GAL6	MBNL1	C1D
CD2AP	FNDC3A	DDX50	SMCHD1
BBS10	ACTR6	MST4	SLMO2
YIPF4	GNG10	PPP1CB	RANBP2
FMR1	ACN9	NUPL1	UBA3
DENND4A	PDZD8	ARFIP1	ACAP2
KRCC1	PELI1	BAZ1A	CAPZA2
PDE8A	RPS3A /// SNORD73A	TAB2	RALGAPA1
RAB11FIP2	PCMTD2	CKS2	CBR4
KIF2A	LOC145783 /// ZNF280D	VCAN	MEX3C
LBR	EMC2	PDE6D	CCNT2
CASP8AP2	TAX1BP1	APPL1	IGJ
APIP	PTGS2	HSDL2	P2RY14
MFN1	IL6ST	MME	ITGA4
SECISBP2L	ITSN2	CSGALNACT2	PTX3
TMEM123	MATR3 /// SNHG4	CUL4B	PSMA3
AGL	SLC38A2	LYPLA1	
**Under-expressed genes**			
HLA-DQA1 /// LOC100509457	C2CD2L	ARID1A	UBE2O
DKK2	RP4-621B10.8	TBL1X	GP1BB /// SEPT5 /// SEPT5-GP1BB
DCHS1	CNTN6	TECR	AKAP13
FMO3	SEMA3E	NENF	RGS10
AF198444	EPB42	CTSZ	ADRA2A
RFPL1	TCF3	ZNF652	DNAJB2
PLEKHM1	ANKRD7	SRPX2	SLC25A31
RGS13	ZIC4	SLC4A8	IGF1R
APBA1	CLIC3	RPL14	LPA
TTC27	MAFK	VPS39	CACNG4
RAD51	LRRC17	SF3A2	PTOV1
OR7E156P	HAPLN2	PDGFA	DDX24
PER2	COPA	CWH43	ASAP3
MAGEB1	NFIC	RPGRIP1L	IGHA1 /// IGHG1 /// IGHM
ADORA2A /// SPECC1L-ADORA2A	CLU	KCNJ5	PDZD2
RGS11	GPD1	PBXIP1	ITGB5
PLXNB2	RXRA	CFB	BRPF1
CDKN1C	AKR1B10	RPAIN	FKBP8
PRB1	LDLR	NFIB	DYNC1LI2
PARVB	PRLR	LOC100506282	TMPRSS5
IGHG1 /// IGHM	RABL2A /// RABL2B	GOSR1	TLL2
CLCA3P	ASCC2	CTRB1 /// CTRB2	TPI1
PRPF6	PP14571	PNPLA2	ANKZF1

CPS1-IT1	ANK1	HSPB8	CCDC85B
NUDC	BMP7	CLTB	ACTR3P2 /// ACTR3P2
BTBD18	TPM2	ALDOB	LARGE
PVALB	VAMP2	F5	RP11-15P13.1
SHARPIN	GAP43	HLA-DOA	MAT1A
PARD3	MGMT	FOXO3	FGFR2
TMSB4Y	GPER1	POU3F2	CACNA1E
MYOD1	NRGN	TSSC4	MSH5-SAPCD1 /// SAPCD1
HIST1H4A /// HIST1H4B /// HIST1H4C /// HIST1H4D /// HIST1H4E /// HIST1H4F /// HIST1H4H /// HIST1H4I /// HIST1H4J /// HIST1H4K /// HIST1H4L /// HIST2H4A /// HIST2H4B /// HIST4H4	SCUBE3	WISP1	HYAL1
GPR22	NADSYN1	RPLP2	POLA2
RUNDC3A	CHERP	ADIRF	F2RL3
WNT5A	CYP2B7P	FUT3	TSGA10
HOXA11	CTDSPL	INSR	XYLB
MVB12B	FARSA	FKSG49	TRO
MEA1	LY6E	C14orf1	SULT2A1
LOC100130331	SLCO1A2	CCNT1	GNGT1
TGM2	BRD4	DENND2A	DHPS
MED16	DRP2	SCAND2P	ZBTB7A
CCDC121	DCAKD	STAG3L3	ARL17A /// ARL17B
SPACA1	AKAP1	MSH6	COX5B
EDN3	NEUROD6	MAP1B	MATN1
ARPC4	SLC6A9	SIT1	FZD5
CLPB	CFDP1	SETD4	SCGB2A1
SHCBP1L	ACKR1	LOC101060747 /// PDPK1	AQP3
ABCC6 /// LOC101930322	SLC7A1	DDX49	DHRS2
ESYT1	ASAP1-IT1	ITGB8	PDE6C
LOC101930075 /// NPIPA1 /// NPIPA2 /// NPIPA3 /// NPIPA5 /// NPIPA7 /// NPIPA8 /// PKD1P1	ANGPT2	ACAP1	

Table S3. Over-expressed genes in controls versus HD patients.

Over-expressed genes			
TAF13	NFYC	PRRC2C	RBM5
CDC42EP1	SYT5	EDNRA	HLA-DRB4
CRYBA1	RYR2	GP1BB /// SEPT5 /// SEPT5-GP1BB	FOXD2
PTOV1	IGH /// IGHA1 /// IGHG1 /// IGHG2 /// IGHG3 /// IGHM /// LOC102725526	DLGAP4	DCAKD
PRLR	TRA2B	PTGER3	TBX1
FGA	TRAF3IP1	RECK	ZNF419
NLGN1	ANG	GUCA2B	PCDH8
TPSAB1	EBF2	GIPR	C17orf75
ATXN2L	AAMDC	BMP8A	CPTP
ABHD2	PTPN21	ATP2C1	ZBTB1
CNTN6	GNRHR	DAZ1 /// DAZ2 /// DAZ3 /// DAZ4	RAB3B
MARK2	RAB11B	DDO	KLF4
ANKRD6	GRIN2D	PHKG2	MSR1
ALCAM	SELE	LARGE	UHRF1BP1L
CDKL3	IRX4	NFIC	SLC26A3
ACO2	CD33	SYCP2	BOP1 /// MIR7112
APBB2	CTSZ	COBLL1	DNAJC6
VPS45	SLC13A1	TEX11	SART3
LEPREL4	TNP2	TP73	IQCK
C1orf112	MICA /// MICB	DPYSL4	MAN1A2
MIR1236 /// NELFE	CCDC70	TFAP2B	TBC1D2
CCDC87	BTF3P11 TCEB3	ACOXL	RPL35A
GRB14	MSX2	IGHA1 /// IGHD /// IGHG1 /// IGHG2 /// IGHG3 /// IGHM /// IGHV4-31	GYS2
ABCA11P	FNDC3A	OR10H2	ZNF580
MIR4800 /// MXD4	PRTN3	RAB3A	SYDE1
SP2	POU3F2	TCF7L2	KLHL7
ELMO3	KAZALD1	RSRC1	TK2
ZNF84	HN1	RPGRIP1L	TAS2R10
SNED1	FAM124B	BHMT2	ZNF225
NT5DC2	LRP5L	GPM6B	C16orf95
EDA	UGT1A1 /// UGT1A3 /// UGT1A5 /// UGT1A8 /// UGT1A9	MEOX2	GBX2
MUC7	KIZ	TACR3	PPFIBP2
RPL5 /// SNORA66 /// SNORD21	F5	DDX17	DCAF15
ATXN3	PRSS50	SNX13	DKFZP434L187
CEP104	DAPK3 /// MIR637	UPB1	LRP5
NUP160	PER2	CCHCR1	IPO4
CREBBP	SHMT2	SIRT4	AGER
BCKDHB	KDM6B	VAC14	ICOSLG
PRIM2 /// PRIM2B	SPINK2	SLC22A2	R3HCC1L

FCER2	ZBED8	ZNF259P1 /// ZPR1	MIPEP
PTPN11	VAV2	DOCK9	FAM155A
BAX	THPO	KATNB1	FAM189A1
PEMT	RNF208	EPB41L4A-AS2	TSN
DCLK2	TP63	SLC12A8	LAMB1
PTPRS	SPTBN4	KLHDC8A	MBNL2
CENPQ	NFASC	HSPB1	MECP2
PRPF31	IDUA	AP3D1	SIGLEC8
PSMD11	PPP6R2	EHBP1L1	ZNF208 /// ZNF595 /// ZNF718
CDKN3	PVR	FAF1	NEURL1
PIGO	HPSE2		
**Under-expressed genes**			
HEATR3	ZNF140	ATF2	TMEM176B
BLZF1	MBL2	RP3-391O22.2 /// RPL7P25	QSER1
SLC39A14	SLC4A7	MINOS1P1	INSIG2
SOCS1	FAM134B	HHAT	THSD7A
MRPS18B	DLG1	NRP1	ITGB1
PCCA	LPAR4	PSD3	LOC101929240 /// SNRNP200
CTDSPL	APPBP2	SMPD1	FBXO5
TRIM14	MMD	PLAGL1	C5orf42
ALDH5A1	FOLH1 /// FOLH1B	LOC101926913	ADRA2A
PPP3CB	VIL1	RP1-263J7.1 /// RPL7P27	DOPEY1
PROSC	CLMN	GAB1	FAM120A
PLS3	TRPC2	ISCA1	DAZL
RRP15	TRHDE	MFN2	CD24
DGKA	DNAJC9	SLC7A4	MBD2
IFIT5	KLHL9	GABRB1	MAGEC2
MAP2K5	DMRT1	RNF128	ACSM2A /// ACSM2B
ZNF80	FUS	ERCC6L	CACNA2D1
GNA13	LOC101929036 /// PAH	SSX3	MALL
PCK1	WARS	CAMSAP2	INTS7
TMPO	JAK2	DDX1	PTPLAD1
NUP62	ZBTB18	CAND2	SNAP91
TM4SF1	MRPS15	THBS1	B3GALT1
MS4A2	CEMIP	PCGF1	NCAPG
RRP7A /// RRP7B	SEPT11	MGC2889	ACE2
CIB2	ITGB3	ZMYM6	MDK
APOL1	LOC101930075 /// PKD1	MINPP1	NMNAT2
BAIAP2L2	ZC3H15	TRHR	TEX30
NUDT4 /// NUDT4P1	PCBD1	CDH12	FASTKD2
IPO7	ATP4B	HGF	GABBR1 /// UBD
VPS33B	CPA3	RHOBTB3	ZMYM4
FAHD2A	RNLS	MAGEA3 /// MAGEA6	C5orf22
TTC40	DSCC1	WEE1	CYP2C8
E2F8	CYP2B6 /// CYP2B7P	HDGFRP3	TARP /// TRGC2 /// TRGV9
NCKAP1	RNF144A	ITPR3	KLK15
TTLL7	ALB	RNF19A	PPP2R5A
NAA16	LCE2B	LANCL1	ASAP2
ARMCX5	SH3GL3	PANX1	C6orf106

GHRHR	SEC23B	GPRASP1	CAMKK2
SIPA1L3	EPB41L4B	MRPL52	PCSK2
PAGE1	PRR7	AGPAT3	N4BP1
ITGA6	ITK	DYRK3	GPR162
SEPHS1	MAP4	DYRK1A	NKX2-1
ADORA1	RAB6B	MCM2	TULP4
ERAP1	CDK17	SERP1	PLAT
NETO2	HINT1	FUBP1	HSPH1
ZNF75D	RARG	KPTN	ID2
LOC100996756	STAT1	MIR3656 /// TRAPPC4	ARMC4
PRKAR2B	PGAP2	GCNT1	ATR
RAD1	LMAN1L	NOLC1	RLBP1
SERPINB9	SHC1P1 /// SHC1P1	TRIM22	

Table S4. Over-expressed genes in controls versus Stage 1 HD patients (Year 1).

**Over-expressed genes**			
SDHD	EREG	GABPB1-AS1	DYNC1I2
PHOSPHO2	GPM6A	TFG	NAA30
JUP	MAB21L3	RASGEF1B	CXCL2
CSMD1	ANGPTL1	PLB1	TMEM144
LYZ	TPMT	RP11-305E6.4	HOXB7
CLECL1	ZNF780A	RNASE4	ZNF519
LRRN3	SFSWAP	NAPB	AC005224.2
LINGO2	ZNRD1-AS1	AHI1	DUXAP10 /// LINC01296
UBE2W	TAF1A-AS1	PPARGC1A	HCG11
EFCAB2	SYN2	RPL27A /// SNORA45A	LIN7A
PLSCR1	CLEC4D	LOC102724537	ZNF252P-AS1
MCPH1	BRCA2	LOC101927560 /// LOC101927587	DNM1L
ABHD12B /// MIR4454	PLEKHG1	HSPD1	GIPC2
ST8SIA1	MRPL39	PIAS2	KDM4C
NEB	GNAI1	SLC2A13	SREK1IP1
MUC20	LOC101927380	RP11-1C8.6	TEAD1
PPP2R3A	AK055458	TTC26	RAP2A
TTN	C12orf66	HAVCR1	MEF2A
TMEM156	LINC00304	SLC16A4	PEG10
PLEKHH2	VPS13C	LINC00630	AF086184
TCF4	ALG10 /// ALG10B	LOC100506730	ZGRF1
SEZ6L	CHD7	NOP14-AS1	SYBU
MYO1B	TIMM17A	ZNF655	LOC645513
NETO1	RP11-395I6.3	ZNF254	FBXO3
FLJ32790	KLHL5	MRPS30	CCP110
ZNF611	RP11-140I16.3	WBP2NL	LINC01184 /// LOC101929964
ZNF443	KIF18A	PDE8B	PCNP
KCTD14 /// NDUFC2-KCTD14	CNIH4	BCL2A1	ANKRD32
TTLL5	LOC101060609	OTUD6B-AS1	DNAJC25 /// DNAJC25-GNG10
MON2	PBRM1	MGC57346	ZMAT1
EPB41L5	ZNF354A	TBC1D12	SPRY3

SFT2D1	MAP7D3	RGS1	POLI
HS2ST1	LOC101930630 /// STRC	SMIM8	MASTL
ABCG1	BRWD1	CLDN22 /// WWC2	RP1-193H18.2
HELLS	TANC2	ZNF678	CEBPZOS
SPATS2L	KCNMB3	RP5-1068B5.3	SRSF3
TCRA /// TCRAV5.1a	COL28A1	HINT3	WDR52
FANCM	F8	SCFD1	G0S2
ZBTB11	ELMOD2	NIPSNAP3B	MILR1
NDC80	PMS1	FAT4	EPHA4
DAAM1	NAA15	DNAJC18	TMEM170B
UGT8	SAMD9	ANKRD6	ERV3-1
FAM105A	ZNF763	AC083843.1	BTG1
ANGEL2	MORF4L2	AK097119 /// RP11-73M18.6	PAICS
TMEM38B	DNALI1	N4BP2L2	LOC101930114
MYCL	IMPAD1	DNAJC10	PCSK5
BRAF	ELFN2	LOC286272	C1GALT1
ZIC2	ABCB9	LMO4	LOC102725022
ST6GAL1	TMTC3	VWA5A	BCAS4
TCERG1	RP11-356B19.11	FLJ35816	
**Under-expressed genes**			
RNF182	NKX3-1	IL23R	DEFA4
ITGA2B	MS4A3	ADAM12	HDC
RUNX1T1	ERBB4	PAQR9	DAB1
IQCA1	C1orf116	GUCY1A3	IGF2BP3
ZNF595	ABCG2	TRIM58	LOC653486 /// SCGB1C1
TGFB1I1	SLC35D3	TMEM158	MYL9
DDX11L2	CPA5	CMBL	ABCC13
SLC6A8	PBX1	PEAR1	PROS1
GP5	RORC	MARCH8	SLC6A10P /// SLC6A10PB /// SLC6A8
CACNG6	TREML1	EGF	SLC4A1
ARG1	HPS1	TRAK1	MYZAP
ANK1	IGDCC4	ANKRD9	ITGB3
PHLPP2	LOC101927723	LGALS2	CMTM5
TSPAN7	LIPH	GYPE	BC039537 /// RP11-30L15.6
GSTM1	VIL1	THBS1	SEC14L5
SMIM24	MYLK	FKBP1B	SH3TC2
KLHDC8A	CA2	PTGES	GP1BB /// SEPT5 /// SEPT5-GP1BB
PPP1R1C	IGLC1 /// IGLV9-49 /// IGLV9-49	TPRG1	GPR126
CR1L	GSTM2	SPTB	SYNJ2
CTSE	TNS1	LINC01192	CLU
PTPRM	KLF2	FAM46C	ST6GALNAC1
RUNDC3A	ALOX12	CD101	GYPB
ARL17A /// ARL17B	LOC101927507	HBM	FSTL1
MGLL	LOC100505609	ERBB3	NEDD4L
TBC1D22B	COL5A1	LGALSL	SFN
ELOVL7	GNG11	SOX6	FAXDC2
NTN4	OSBP2	LINC00853	TTC39A

TMCC2	LOC101928554	NFIX	NELL1
ASPM	KCNH2	TMEM107	ZBTB7C
LOC100134445 /// LOC100288778 /// LOC100653296 /// WASH1 /// WASH2P /// WASH3P /// WASH5P /// WASH7P	SLC2A1	WNK1	MICAL2
TGM2	LOC100507530	KEL	HTR2A
RFX2	SPARC	GFI1B	GID4
RGS7BP	LYL1	BC041363	SLC7A5
MDGA1	NINL	AY927499	SESN3
YAP1	AL109706	BCL2L1	LOC340340
PLS3	LY6G6D /// LY6G6F	TFR2	HBD
ABLIM3	LOC283045	EDNRB-AS1	HKDC1
NHLH2	FSBP /// RAD54B	JAZF1	REEP1
GP1BA	HIST1H3C	P2RY12	TSHB
CTTN	TMEM229B	CDH17	TMEM86B
ABCC4	ITGB5	UBAC2-AS1	HOMER2
FAR2	GYPA	SEMG1	LOC93444
TTC7B	DCTN1-AS1	ERC1	C19orf33
PCYT1B	ELL2	TUBB1	TPPP3
GPR146	TSPAN6	EPB41	NFIB
GREM2	YBX3	ADD2	ATRNL1
ABCC3	MAN1A1	LOC100996902	DLX2
ENDOD1	GUCY1B3	LOC101927599	C7orf73 /// LOC101930655
BCAM	PLCB4	ATP1B2	SOX11
ALS2CR12	NPRL3	ZNF385D	

Table S5. Over-expressed genes in controls versus Stage 2 HD patients (Year 3).

**Over-expressed genes**			
BC044596	SDHD	ARHGEF7	APOL6
ZNF417	C1QTNF3	LOC101928623 /// LOC401320	NEK5
MATR3	RPS20 /// SNORD54	IBTK	APBB2
MGC57346	TRMT2B	BCAS4	TREML4
LRRC37BP1	CXorf56	N4BP2L2	GABPB1-AS1
HEATR5B /// HEATR5B	INIP	ABI2	KCNE3
KIAA1257	TBC1D12	RNF125	SUSD4
TMEM165	EPG5	CLEC4C	RNASEH1
ARHGAP26	TFDP2	AHI1	FLJ21369
SPAG9	SLC17A5	FARSB	MRE11A
AKT3	LOC340184	XPNPEP3	LOC101930150 /// NF1
VPS13D	LOC101929910 /// LOC613037 /// NPIPA5 /// NPIPB3 /// NPIPB4 /// NPIPB5	MB21D1	DNAH1
FBXO32	BC048103 /// CSGALNACT1	SCRG1	INTS4
RC3H2	LRRC19	METTL6	SORBS2

AVL9	ALDOAP2	LOC101927550	LOC142937
AX747826	SSTR2	GART	KIAA1430
RALGAPA1	MTMR7	LRRC7	ZFYVE16
SUB1	LILRA3	ATM	LOC101060521 /// POLR3E
THOC5	RSU1	KRTAP5-AS1	MDM4
ALB	HIPK2	APPBP2	P4HA3
CFDP1	LOC283693	MGC16142	DYNC2LI1
PBRM1	CEBPZOS	PAPOLA	ARID4B
FAM153A /// FAM153B /// LOC100507387	BMPR1A	AREG	SFT2D1
AX748294 /// RP11-1006G14.1	AX747730	MCM4	LOC101928767
CCBL1	CCND3	ACSL4	PRSS23
CALML4	ACTA2	SLC25A42	TRAPPC2
OSER1-AS1	TMPRSS3	LOC100996902	SKIL
IPP	GLRX3	TECR	ARNT
ZNF780A /// ZNF780B	OTUD7B	LINC00467	KIAA1377
GFM1	LOC100506476	C10orf25	PHOSPHO2
GPR85	CTC-444N24.11	LOC100507053	NHLRC2
SNX29	CBY3	CPEB3	RCL1
HSDL2	FTX	TMEM150C	ACP1
PLEKHA2	ARV1	CATSPERB	TAS2R4
TSR1	ZDHHC9	CYP51A1 /// LRRD1	XRCC5
PNMA3	TEP1	MROH1	SNRPN /// SNURF
DSERG1	DISP1	SMKR1	SLC9A1
ZMYND8	CDH4	NUP54	CPM
RP11-73M18.7	ZNF138	SLC28A2	ITIH2
BC039537 /// RP11-30L15.6	POLR1E	CTD-2310F14.1	ZNF790-AS1
TF	PTK2	DIP2C	THBS1
HTR2A	TAF1A-AS1	XKR6	DCAF8
CEP152	PPM1K	MYO9A	PPWD1
CHDH	TTLL5	IL31RA	PDXDC1
RP11-210K20.4 /// RP11-452K12.7	SRGAP1	ERICH1 /// FLJ00290	PRCD
ANKH	CCNC	RREB1	ZNRD1-AS1
JMY	RPS2P45 /// RPS2P45	PPP1R16B	HIVEP3
CYP3A5	C5orf28	CASK	LOC732360 /// TDG
IFT80	LOC101927204	LOC100129518 /// SOD2	LOC283922 /// PDPR
KLHL10	DUSP2	EPHA4	
**Under-expressed genes**			
RNF182	APOBEC3B	OLFM4	CD177
MS4A3	CEACAM6	C17orf97	ERG
CTSG	RNASE3	IGF2 /// INS-IGF2	DEFA4
OLR1	ZNF595	PRSS33	MMP8
GYPA	ELANE	TMEM17	SESN3
SLC22A16	ASPM	SHOX2	PGM5
NFIB	CYP19A1	ENPP3	CRISP2
AZU1	LUM	PAQR9	OLIG2

TRIM51	ITLN1	PCDH9	GJB6
ABCC13	C8orf88	KANK2	DENND1B
LOC101927507	SOX7	DPY19L1	ADRB1
ELL2	LINC00664	GNG11	RP11-38P22.2
KIAA0101	IPO11 /// LRRC70	SSFA2	MYCT1
CCDC176	CTSE	DNAJC6	ABCA13
NPR3	HOTS	NID2	LOC100996668 /// ZEB1
PLOD2	AP000253.1	ADAM12	MANEA
LOC100506098	PPP1R17	FKBP1B	SMIM24
RP11-382F24.1 /// RP11-382F24.2	PROS1	GUCY1B3	HBBP1
TBC1D22B	WDR11-AS1	SLC6A8	MPO
SPARC	SIAE	DAB1	RAB27B
ARHGEF12	LOC100653086	PLD1	ZNF208 /// ZNF595 /// ZNF718
CLIC2	LAIR2	RP11-496I2.2	CCDC15
NEBL	NPHP1	HEPH	BEX1
LOC101927599	TSPAN2	HPGD	BPGM
DLX6	SLC6A10P /// SLC6A10PB /// SLC6A8	RNF11	NARF
CES1 /// LOC100653057	TRIM58	LEPR /// LEPROT	HEMGN
EPB41	BIVM	YOD1	ELMOD2
SVOPL	COL6A3	NUDT4 /// NUDT4P1 /// NUDT4P2	CYP1B1
MPP7	ZNF677	LIN7C	TMCC2
SLC2A1	NUDCD1	OSBP2	HBM
BCL2L1	GALNT5	USP6NL /// USP6NL-IT1	WWC1
TC2N	CNKSR3	FSBP /// RAD54B	HBD
BC016361	ABCC4	ZNF385C	CYP4F12
FRMD4A	TNS1	SORBS1	COMMD8
MSRB3	NCKAP1	ANK1	RETN
SSX2IP	HPS1	XPO7	FLJ22763
MEIS3P1	DACH1	CCDC126	DLX2
ARHGAP6	WDHD1	KIAA1024	KLHL20
OTUD6B-AS1	CAMP	FKBP1B /// MFSD2B	MAP6D1
C2orf49	CDCA5	IGLC1 /// IGLV9-49 /// IGLV9-49	SLC24A3
PRKG1	SPATA5	CDH17	FAXDC2
CEBPE	CD1E	KEL	RHOBTB3
RP11-672L10.6	SLITRK4	MARCH8	PTGFRN
RNF212B	TRIQK	SOX6	ARG1
TGM2	RNF14	LINC01448	F2RL1
CENPBD1P1 /// RPL23AP7	PEG3	DIS3L2	ZNF165
TCP11L2	TAF6L	RIOK3	FAR2
TEX11	RHOU	MARCH6	RP11-157E16.1
GUCY1A3	ZNF702P	TBCEL	TRIM23
RP11-61L19.3	KAT2B	C7orf73 /// LOC101930655	

Table S6. The top-15 ranked pathways obtained from Metascape for pre-symptomatic HD using KEGG.

Rank	Pathway Name	*Log10 (p-value)*
1	Bile secretion	-15.132
2	Focal adhesion	-11.883
3	Aldosterone synthesis and secretion	-8.821
4	Inflammatory bowel disease	-7.912
5	Crushing syndrome	-7.621
6	Hippo signaling pathway	-7.588
7	Dilated cardiomyopathy	-5.331
8	Endocytosis	-2.788
9	Huntington disease	-2.739
10	Neurotrophin signaling pathway	-2.035
11	Homologous recombination	-2.018
12	Lysosome	-1.980
13	Complement and coagulation cascade	-1.861
14	Axon guidance	-1.856
15	Cocaine addiction	-1.807

Table S7. The top-15 ranked pathways obtained from Metascape for pre-symptomatic HD using GO-Biological Processes, Molecular Function and Cellular Components.

Rank	Pathway Name	*Log10 (p-value)*
	**GO-Biological Processes**	
1	Embryonic morphogenesis	-14.230
2	Cell morphogenesis	-13.303
3	Behavior	-10.949
4	Response to alcohol	-10.743
5	Negative regulation of cell differentiation	-10.541
6	Brain development	-9.889
7	Regulation of kinase activity	-9.638
8	Cellular glucuronidation	-9.427
9	Regulation of neuron death	-9.229
10	Response to hormone	-8.998
11	Cellular response to nitrogen compound	-8.759
12	Eye development	-8.494
13	Apoptotic signalling pathway	-8.462
14	Regulation of apoptotic signaling pathway	-8.377
15	Growth	-8.000

**GO-Molecular Functions**		
1	Glucuronosyltransferase activity	-15.111
2	Protein kinase activity	-8.591
3	Transcription coactivator binding	-7.942
4	Lysine acetylated histone binding	-7.169
5	Collagen binding	-6.987
6	SMAD binding	-6.649
7	Transcription factor binding	-6.262
8	Immunoglobulin receptor binding	-6.241
9	Beta-tubulin binding	-6.165
10	Calcium channel regulator activity	-5.633
11	Floppase activity	-5.575
12	2-oxoglutarate dependent dioxygenase activity	-5.508
13	Proline-rich region binding	-5.491
14	Chromatin binding	-5.365
15	Phosphatidylinositol binding	-4.458
**GO-Cellular Components**		
1	Synaptic membrane	-11.360
2	Basement membrane	-10.602
3	Post-synapse	-10.182
4	Neuromuscular junction	-9.099
5	Coated vesicle	-7.984
6	Inclusion body	-7.213
7	Site of double-strand break	-7.097
8	Perinuclear region of cytoplasm	-7.005
9	Transcription regulator complex	-6.716
10	Rough Endoplasmic reticulum membrane	-5.303
11	Neuronal cell body	-5.270
12	Focal adhesion	-4.859
13	Nuclear envelope	-4.084
14	Early endosome	-3.703
15	Mitochondrial matrix	-3.620

Table S8. The top-15 ranked pathways obtained from Metascape for symptomatic HD using KEGG.

Rank	Pathway Name	*Log10 (p-value)*
1	Alcoholism	-11.414
2	Endocytosis	-6.798
3	Starch and sucrose metabolism	-4.943
4	Transcriptional mis-regulation in cancer	-4.604
5	PI3K-Akt signalling pathway	-4.213
6	Pathways in cancer	-4.006
7	Regulation of actin cytoskeleton	-3.291
8	NOD-like receptor signalling pathway	-2.812
9	Proteoglycans in cancer	-2.527
10	Alzheimer disease	-2.403
11	Leishmaniasis	-2.297
12	Toxoplasmosis	-2.249
13	Ovarian steroidogenesis	-2.212
14	Coronavirus disease-COVID-19	-2.179
15	Crushing syndrome	-2.141

Table S9. The top-15 ranked pathways obtained from Metascape for symptomatic HD using GO-Biological Processes, Molecular Function and Cellular Components.

Rank	Pathway Name	*Log10 (p-value)*
	**GO-Biological Processes**	
1	Protein localization to CENP-A containing chromatin	-28.140
2	Response to hormone	-10.590
3	Regulation of cell cycle process	-10.209
4	Regulation of kinase activity	-9.716
5	Growth	-9.610
6	Membrane organization	-9.340
7	Placenta development	-8.893
8	Carbohydrate metabolic process	-8.720
9	Regulation of cell activation	-8.076
10	Cellular response to lipid	-7.941
11	Negative regulation of cell population proliferation	-7.524
12	Hemopoiesis	-7.279
13	Protein catabolic process	-7.120
14	Cell morphogenesis involved in neuron differentiation	-6.823
15	Amyloid-beta clearance	-6.743
	**GO-Molecular Functions**	

1	Structural constituent of chromatic	-20.647
2	Alpha-amylase activity	-13.082
3	Immunoglobulin receptor binding	-8.136
4	Amyloid-beta binding	-7.143
5	Ubiquitin binding	-6.881
6	Cyclin dependent protein serine/threonine kinase regulator activity	-6.794
7	Phospholipid binding	-6.569
8	Protein carrier chaperone	-5.978
9	Virus receptor activity	-5.847
10	Phospholipid transporter activity	-4.989
11	Transcription factor binding	-4.986
12	Chromatin binding	-4.881
13	Insulin binding	-4.799
14	G-protein beta-subunit binding	-4.658
15	Histone H4 acetyltransferase activity	-4.658
	**GO-Cellular Components**	
1	CENP-A containing nucleosome	-32.950
2	Coated vesicle	-14.202
3	Site of double-strand break	-12.094
4	SWI/SNF superfamily-type complex	-9.752
5	Inclusion body	-8.883
6	Membrane coat	-8.213
7	Transcription repressor complex	-7.134
8	Transcription regulator complex	-6.685
9	Pre-synaptic membrane	-6.478
10	Immunoglobulin complex, circulating	-6.415
11	Golgi membrane	-6.073
12	Perinuclear region of cytoplasm	-5.221
13	Endosome membrane	-5.122
14	Organelle membrane contact site	-4.974
15	Neuronal cell body	-4.863

Table S10. The top-15 ranked pathways obtained from Metascape for controls versus HD patients using KEGG.

Rank	Pathway Name	*Log10 (p-value)*
1	Hematopoietic cell lineage	-12.885
2	Retinol metabolism	-12.058
3	Arrhythmogenic right ventricular cardiomyopathy	-9.625
4	Toxoplasmosis	-8.633
5	Butanoate metabolism	-5.465
6	Glyoxylate and dicarboxylate metabolism	-5.324
7	Cell adhesion molecules	-4.102
8	Neuroactive ligand-receptor interaction	-4.070
9	Glucagon signaling pathway	-3.659
10	Human T-cell leukemia virus 1 infection	-3.629
11	Proteoglycans in cancer	-3.240
12	Oxytocin signaling pathway	-3.197
13	Phospholipase D signaling pathway	-2.818
14	Sphingolipid signaling pathway	-2.624
15	Pathways of neurodegeneration-multiple diseases	-2.491

Table S11. The top-15 ranked pathways obtained from Metascape for controls versus HD patients using GO-Biological Processes, Molecular Function and Cellular Components.

Rank	Pathway Name	*Log10 (p-value)*
	**GO-Biological Processes**	
1	Gland development	-20.375
2	Regulation of hormone levels	-17.274
3	Tube morphogenesis	-14.716
4	Response to hormone	-12.197
5	Response to wounding	-12.136
6	Regulation of kinase activity	-10.575
7	Circulatory system process	-10.329
8	Tissue morphogenesis	-10.141
9	Neuron projection development	-9.327
10	Negative regulation of cell population proliferation	-9.038
11	Regulation of vesicle-mediated transport	-8.967
12	Appendage development	-8.740
13	Regulation of cytoskeleton organization	-8.308
14	Regulation of proteolysis	-8.207

15	Positive regulation of cell motility	-8.161
	**GO-Molecular Function**	
1	Carboxylic acid binding	-11.837
2	Cell adhesion molecule binding	-9.114
3	Translation activator activity	-7.856
4	Myosin V binding	-7.598
5	Co-receptor activity	-7.376
6	Amide Binding	-7.081
7	Protein kinase binding	-6.777
8	Promoter-specific chromatin binding	-6.773
9	Immunoglobulin receptor binding	-6.051
10	Protein tyrosine phosphatase activity	-5.971
11	Non -membrane spanning protein kinase activity	5.784
12	Exopeptidase activity	-5.725
13	Molecular adaptor activity	-5.587
14	Cell adhesion mediator activity	-5.141
15	Sialic acid binding	-4.950
	**GO-Cellular Components**	
1	Post-synapse	-11.188
2	Platelet alpha granule	-10.203
3	IgG immunoglobulin complex	-9.086
4	Axon	-7.698
5	Protein complex involved in cell adhesion	-7.686
6	Main axon	-7.428
7	Pre-synapse	-7.276
8	External side of plasma membrane	-5.468
9	Condensed nuclear chromosome	-5.241
10	Cajal body	-5.220
11	Neuronal dense core vesicle	-5.034
12	Nuclear outer membrane	-4.942
13	Golgi membrane	-4.195
14	Dendrite	-4.062
15	Mitochondrial matrix	-3.756

Table S12. The top-15 ranked pathways obtained from Metascape for controls versus Stage 1 (Year 1) HD patients using KEGG.

Rank	Pathway Name	*Log10 (p-value)*
1	Platelet activation	-15.021
2	Gap junction	-10.535
3	ABC transporters	-8.375
4	ErbB signalling pathway	-6.963
5	N-Glycan biosynthesis	-6.190
6	Hippo signaling pathway-multiple species	-5.081
7	Malaria	-4.360
8	Transcriptional mis-regulation in cancer	-3.038
9	p53 signalling pathway	-2.513
10	Aldosterone-regulated sodium reabsorption	-2.465
11	Complement and coagulation cascade	-2.259
12	Insulin secretion	-2.259
13	Fluid shear stress and atherosclerosis	2.234
14	Ether lipid metabolism	-2.097
15	Fanconi anemia pathway	-2.005

Table S13. The top-15 ranked pathways obtained from Metascape for controls versus Stage 1 (Year 1) HD patients using GO of Biological Processes, Molecular Function and Cellular Components.

Rank	Pathway Name	*Log10 (p-value)*
	**GO-Biological Processes**	
1	Regulation of body fluid levels	-11.617
2	Regulation of sequestering of calcium ion	-11.031
3	Circulatory system process	-10.625
4	Actin filament-based process	-9.575
5	Apoptotic mitochondrial changes	-9.164
6	Tube morphogenesis	-8.810
7	Tissue morphogenesis	-8.792
8	Cell activation	-8.511
9	Cellular response to xenobiotic stimulus	-8.210
10	Sex differentiation	-7.934
11	Actomyosin structure organization	-7.299
12	Positive regulation of kinase activity	-7.153
13	Cell population proliferation	-6.743
14	Xenobiotic transport	-6.707

15	Cellular response to organonitrogen compound	-6.697
	**GO-Molecular Function**	
1	Extracellular matrix binding	-12.353
2	Virus receptor activity	-9.306
3	ABC-type transporter activity	-8.416
4	Epidermal growth factor receptor binding	-7.231
5	Protein homodimerization activity	-6.902
6	Actin binding	-6.784
7	Oxidoreductase activity, acting on peroxide as acceptor	-6.142
8	Spectrin binding	-5.364
9	Calcium ion binding	-4.643
10	DNA-binding transcription factor binding	-4.272
11	Ribonucleoside triphosphate phosphatase activity	-4.030
12	Ubiquitin protein ligase binding	-3.634
13	Dynein heavy chain binding	-3.619
14	Sialyltransferase activity	-3.169
15	Lysophospholipase activity	-2.99
	**GO-Cellular Components**	
1	Platelet alpha granule	-14.569
2	Intercellular bridge	-8.776
3	Platelet alpha granule membrane	-8.252
4	Side of membrane	-8.229
5	Actin cytoskeletion	-8.006
6	Glycoprotein Ib-IX-V complex	-7.700
7	Sacroplasmic reticulum	-7.410
8	Spectrin associated cytoskeleton	-6.778
9	Cell cortex	-5.014
10	Actin-based cell projection	-4.424
11	Specific granule lumen	-4.145
12	Photoreceptor inner segment	-3.969
13	Golgi lumen	-3.575
14	Lamellipodium	-3.514
15	Pre-synapse	-3.511

Table S14. The top-12 ranked pathways obtained from Metascape for controls versus Stage 2 (Year 3) HD patients using KEGG.

Rank	Pathway Name	*Log10 (p-value)*
1	Salivary secretion	-11.698
2	p53 signaling pathway	-9.848
3	Transcriptional mis-regulation in cancer	-3.804
4	Chemical carcinogenesis-reactive oxygen species	-3.381
5	Proteoglycans in cancer	-2.625
6	Adipocytokine signaling pathway	-2.661
7	Homologous recombination	-2.383
8	cAMP signaling pathway	-2.040
9	Steroid hormone biosynthesis	-1.885
10	Viral life cycle - HIV-1	-1866
11	Neutrophil extracellular trap formation	-1.752
12	Amoebiasis	-1.371

Table S15. The top-15 ranked pathways obtained from Metascape for controls versus Stage 2 (Year 3) HD patients using GO of Biological Processes, Molecular Function and Cellular Components.

Rank	Pathway Name	*Log10 (p-value)*
	**GO-Biological Processes**	
1	Circulatory system process	-10.399
2	Negative regulation of cell population proliferation	-9.736
3	Regulation of epithelial cell proliferation	-9.013
4	Cellular response to nitrogen compound	-8.920
5	Positive regulation of phosphorylation	-8.762
6	Regulation of cell-substrate adhesion	-8.151
7	Antibacterial humoral response	-8.016
8	Cellular response to transforming growth factor beta stimulus	-7.982
9	Response to oxygen levels	-7.929
10	Regulation of cell activation	-7.825
11	Regulation of smooth muscle cell proliferation	-7.679
12	Positive regulation of reactive oxygen species metabolic process	-7.516
13	Response to toxic substance	-6.930

14	Inorganic ion homeostasis	-6.702
15	Regulation of muscle system process	-6.454
	**GO-Molecular Function**	
1	Oxygen binding	-9.339
2	Manganese ion binding	-8.190
3	Antioxidant activity	-7.677
4	Oxidoreductase activity	-6.843
5	DNA helicase activity	-6.430
6	Cytoskeletal anchor activity	-5.708
7	Molecular carrier activity	-5.699
8	Protein tyrosine phosphatase activity	-5.640
9	GTPase regulator activity	-3.663
10	Peptide transmembrane transporter activity	-3.655
11	Kinase binding	-3.236
12	Protease binding	-3.032
13	Protein domain specific binding	-2.842
14	Catalytic activity, acting on a nucleic acid	-2.817
15	ubiquitin conjugating enzyme binding	-2.702
	**GO-Cellular Components**	
1	Secretory granule lumen	-23.247
2	Actomyosin	-10.449
3	Tertiary granule	-10.264
4	Platelet alpha granule lumen	-8.624
5	Adherens junction	-6.497
6	collagen-containing extracellular matrix	-6.033
7	Basal part of cell	-4.689
8	Focal adhesion	-4.406
9	Apical plasma membrane	-4.201
10	Nuclear	-3.249
11	Blood microparticle	-2.964
12	Centrosome	-2.742
13	Plasma membrane raft	-2.646
14	Chromosome, telomeric region	-2.611
15	Ciliary transition zone	-2.596

Table S16. 128 common gene targets of HD among CTD, Malacards and Disease Databases

128 common gene targets			
MAOB	MAOA	HTT	CNR1
RCAN1	OGG1	PRNP	GDNF
APP	DRD1	DRD2	BDNF
CASP3	SOD1	NFKB1	MAP2
SIRT1	CASP8	SNCA	TP53
PPARGC1A	SQSTM1	SLC6A3	VEGFA
CREB1	CYCS	CP	MAPT
TH	TGFB1	CREBBP	ACHE
PARP1	NOS1	PPP1R1B	PPARG
BECN1	GSR	CASP1	GRIN1
HAAO	SP1	IGF1	SERPINI1
CPLX2	NGF	SUMO1	GAPDH
CHAT	TARDBP	PSEN1	FEN1
GRIN2A	ENO2	FGFR3	TSPO
AR	ITPR1	GRIN2B	NPY
ATF6	FOXO3	CNTF	HSP90AA1
ZDHHC13	GRM3	HAP1	IFT57
RASD2	FMR1	HDAC9	KMO
ATG7	FOXP1	CACNA1A	CALB2
NEAT1	TGM1	PDYN	MTHFR
XPC	CKB	SLC2A3	HSPA4
TGM2	HDAC1	SLC1A2	GRIK2
NTF3	SYNDIG1L	SLC2A4RG	CARD16
PDE6B	GRIK1	SETDB1	IDUA
BCL11B	HCRT	GRM2	FAN1
FOLH1	NPY2R	MTDH	KALRN
TCERG1	TAF1	PCSK2	PDE10A
JPH3	ZNF395	PCM1	ATXN1
CALB1	RPS27A	AADAT	GRM1
MSX1	DFFB	ATXN3	VPS13A
PCSK1	PFN1	UBE3A	PSEN2
NDUFS4	ALDH7A1	ERCC6	UCHL1

Table S17. MCODE cluster analysis of the 128 common genes between the three public databases.

Gene name	Node Status	MCODE Score	MCODE Clusters
CYCS	14.65	Seed	1
SOD1	14.65	Clustered	1
CASP3	14.65	Clustered	1
APP	14.65	Clustered	1
SIRT1	14.65	Clustered	1
HSP90AA1	14.03	Clustered	1
ATG7	13.76	Clustered	1
SQSTM1	13.76	Clustered	1
PPARG	13.57	Clustered	1
HTT	13.56	Clustered	1
BECN1	13.24	Clustered	1
CASP8	13.17	Clustered	1
HSPA4	12.74	Clustered	1
TP53	12.72	Clustered	1
GAPDH	12.72	Clustered	1
FOXO3	12.29	Clustered	1
IGF1	11.89	Clustered	1
CASP1	11.78	Clustered	1
VEGFA	11.73	Clustered	1
PSEN1	11.35	Seed	2
HDAC1	11.32	Clustered	2
CHAT	10.81	Clustered	2
MAP2	10.66	Clustered	2
PP1R1B	10.57	Clustered	2
ENO2	10.39	Clustered	2
CREBBP	10.35	Clustered	2
PARP1	10.24	Clustered	2
TH	10.19	Clustered	2
NFKB1	10.17	Clustered	2
PPARGC1A	10.74	Clustered	2
SNCA	10.03	Clustered	2
GDNF	10.01	Clustered	2
GRIN2B	9.97	Clustered	2
AR	9.60	Clustered	2
SLC6A3	9.59	Clustered	2
NGF	9.53	Clustered	2
CNR1	9.35	Clustered	2
GRM3	9.35	Clustered	2
GRM1	9.35	Clustered	2
PRNP	9.35	Clustered	2
SLC1A2	9.34	Clustered	2
ATF6	9.27	Clustered	2
BDNF	9.23	Clustered	2
GSR	9.13	Clustered	2
PDYN	9.12	Clustered	2

UCHL1	9	Unclustered	3
TGFB1	9	Seed	3
PSEN1	8.98	Clustered	3
CREB1	8.85	Clustered	3
SP1	8.85	Clustered	3
DRD1	8.83	Clustered	3
GRM2	8.59	Clustered	3
TARDBP	8.57	Clustered	3
MAPT	8.51	Clustered	3
DRD2	8.39	Clustered	3
GRIN2A	8.19	Clustered	3
NTF3	8.18	Clustered	3
CALB2	8.16	Clustered	3
GRIN1	8.04	Clustered	3
CALB1	8.0	Unclustered	3
GRIK2	8.0	Clustered	3
HDAC9	8.0	Clustered	3
HCRT	8.0	Clustered	3
GRIK1	8.0	Clustered	3
ATXN1	7.95	Clustered	3
ACHE	7.90	Clustered	3
NPY	7.64	Clustered	3
ITPR1	7.62	Clustered	3
CNTF	7.27	Clustered	3
MAOB	7.0	Unclustered	-
TGM2	6.97	Unclustered	-
FMR1	6.86	Clustered	-
NOS1	6.41	Clustered	5
RPS27A	6.37	Unclustered	-
MAOA	6.3	Clustered	5
SUMO1	6.0	Unclustered	-
CACNA1A	6.0	Unclustered	-
DFFB	6.0	Unclustered	-
BCL11B	5.78	Unclustered	-
MTHFR	5.78	Unclustered	-
FGFR3	5.64	Clustered	5
ATXN3	5.0	Seed	4
XPC	5.0	Clustered	4
ERCC6	4.76	Unclustered	-
PFN1	4.76	Unclustered	-
SLC2A3	4.76	Unclustered	-
SETDB1	4.63	Unclustered	-
UBE3A	4.52	Unclustered	-
PDE10A	4.28	Clustered	4
FEN1	4.28	Clustered	4
OGG1	4.0	Unclustered	-
TAF1	4.0	Unclustered	-

NDUFS4	4.0	Unclustered	-
RCAN1	3.73	Unclustered	-
RASD2	3.73	Unclustered	-
ALDH7A1	3.73	Unclustered	-
PCSK1	3.0	Unclustered	-
KALRN	3.0	Unclustered	-
CPLX2	3.0	Unclustered	-
TSPO	3.0	Unclustered	-
FOXP1	3.0	Unclustered	-
MSX1	3.0	Unclustered	-
CKB	2.7	Unclustered	-
CP	2.40	Unclustered	-
HAP1	2.0	Unclustered	-
JPH3	2.0	Unclustered	-
FOLH1	2.0	Unclustered	-
PCSK2	2.0	Unclustered	-
CARD16	2.0	Unclustered	-
NPY2R	2.0	Unclustered	-
TCERG1	2.0	Unclustered	-
KMO	2.0	Unclustered	-
PCM1	2.0	Unclustered	-
HAAO	1.6	Unclustered	-
IFT57	1.2	Unclustered	-
AADAT	1.06	Unclustered	-
VPS13A	0.6	Unclustered	-
ZNF395	0.6	Unclustered	-
MTDH	0.5	Unclustered	-
IDUA	0.4	Unclustered	-
PDE6B	0.0	Unclustered	-
SERPINI1	0.0	Unclustered	-
SYNDIG1L	0.0	Unclustered	-
SLC2A4RG	0.0	Unclustered	-
ZDHHC13	0.0	Unclustered	-
FAN1	0.0	Unclustered	-
TGM1	0.0	Unclustered	-

Table S18. Genes for Cluster 1 using MCODE.

Gene	MCODE Score
CASP3	14.65
APP	14.65
SIRT1	14.65
CYCS	14.65
SOD1	14.65
HSP0AA1	14.03
ATG7	13.76
SQSTM1	13.76

PPARG	13.57
HTT	13.56
BECN1	13.24
CASP8	13.17
HSPA4	12.74
GAPDH	12.72
TP53	12.72
FOXO3	12.29
IGF1	11.89
CASP1	11.78
VEGFA	11.73

Table S19. Genes for Cluster 2 using MCODE.

Gene	MCODE Score
UCHL1	9.12
PDYN	9.13
GSR	9.23
BDNF	9.27
ATF6	9.34
SLC1A2	9.35
GRM3	9.35
GRM1	9.35
PRNP	9.35
CNR1	9.53
NGF	9.59
SLC6A3	9.60
AR	9.97
GRIN2B	10.01
GDNF	10.03
SNCA	10.07
PPARGC1A	10.17
NFKB1	10.19
TH	10.24
PARP1	10.24
CREBBP	10.35
ENO2	10.39
PPP1R1B	10.57
MAP2	10.66
CHAT	10.81
HDAC1	11.32
PSEN	11.35

Table S20. Genes for Cluster 3 using MCODE.

Gene	MCODE Score
PSEN2	9.0
CREB1	8.98
DRD1	8.85
GRM2	8.83
TARDBP	8.59
MAPT	8.57
DRD2	8.51
GRIN2A	8.39
NFT3	8.19
CALB2	8.18
GRIN1	8.16
CALB1	8.04
GRIK2	8.0
HCRT	8.0
ATXN1	8.0
GRIK1	8.0
ACHE	7.95
NPY	7.90
ITPR1	7.64
CNTF	7.62
MAOB	7.27

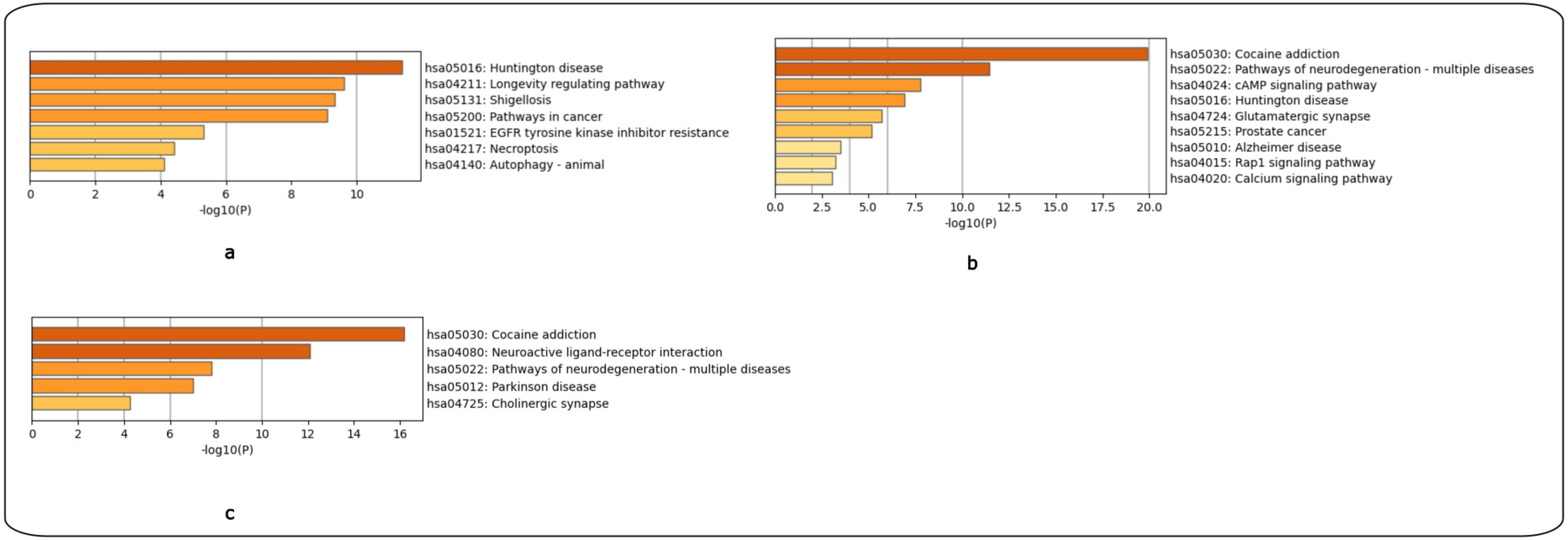

Figure S1. MCODE gene modules KEGG analysis Metascape. (a) MCODE Cluster 1 pathway analysis, (b) MCODE Cluster 2 pathway analysis and (c) MCODE Cluster 3 pathway analysis.

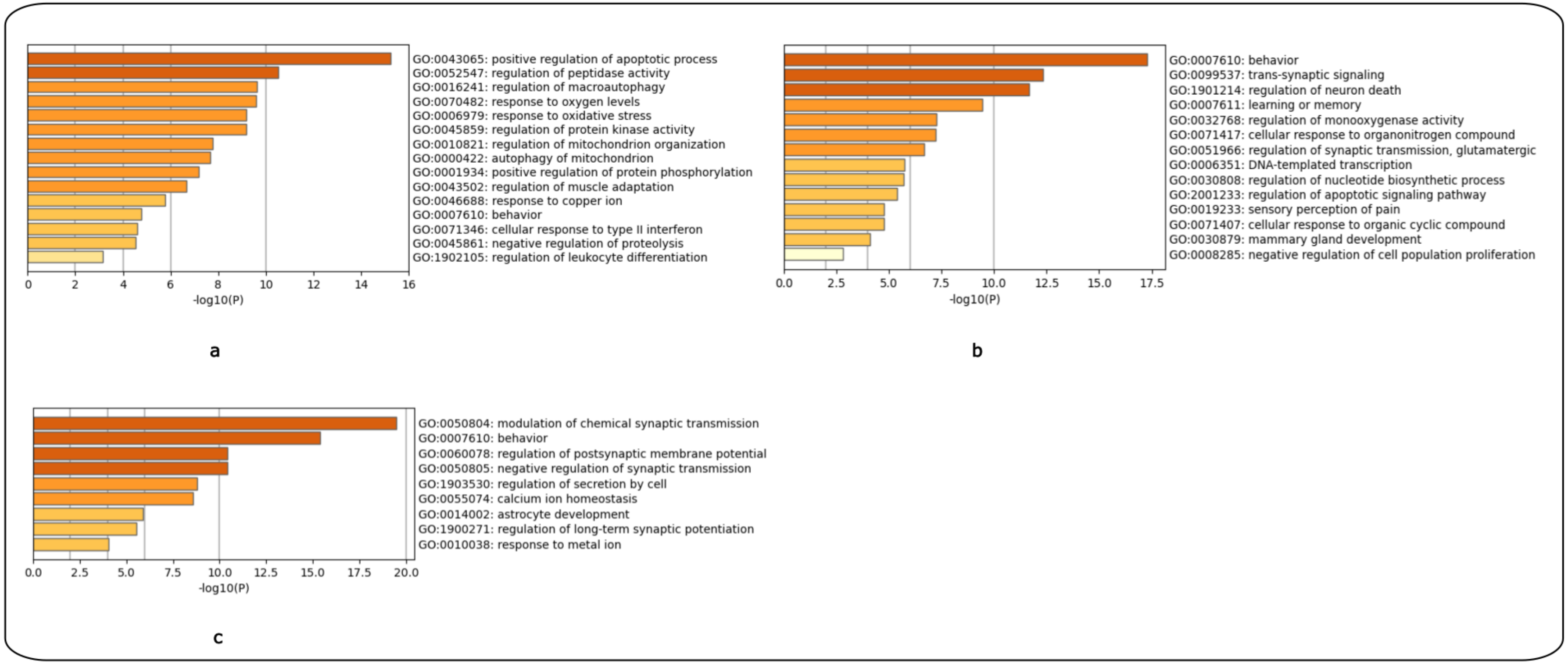

Figure S2. MCODE gene modules GO-Biological Process analysis using Metascape. (a) MCODE Cluster 1 GO analysis, (b) MCODE Cluster 2 GO and (c) MCODE Cluster 3 GO.

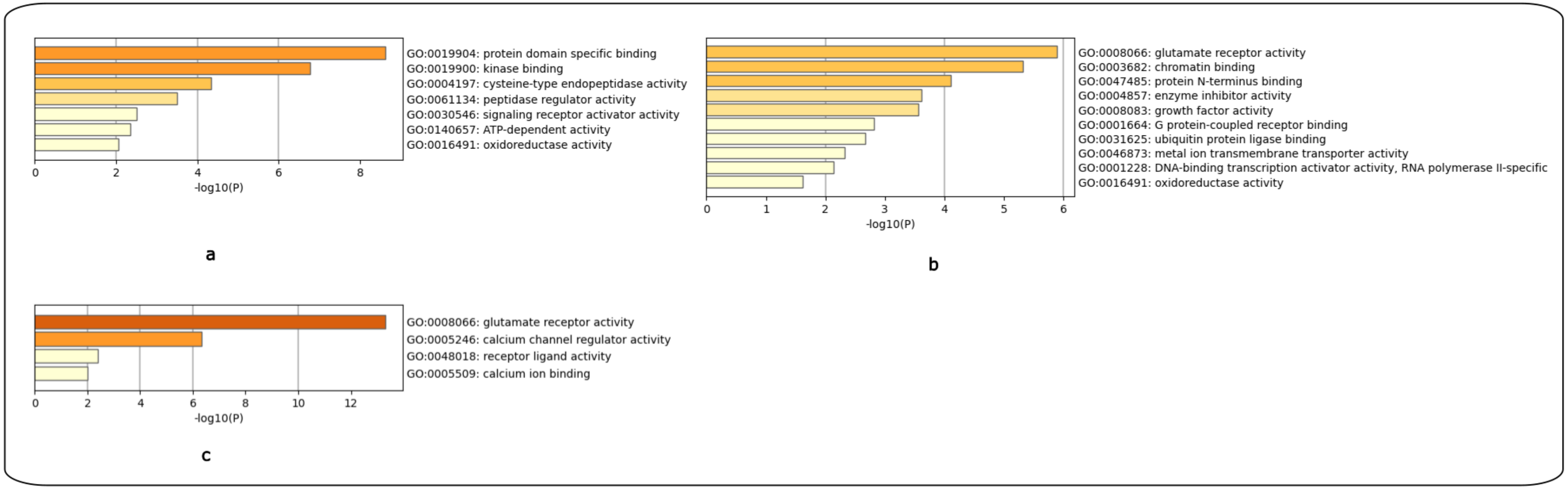

Figure S3. MCODE gene modules GO-Molecular Function analysis using Metascape. (a) MCODE Cluster 1 GO analysis, (b) MCODE Cluster 2 GO and (c) MCODE Cluster 3 GO.

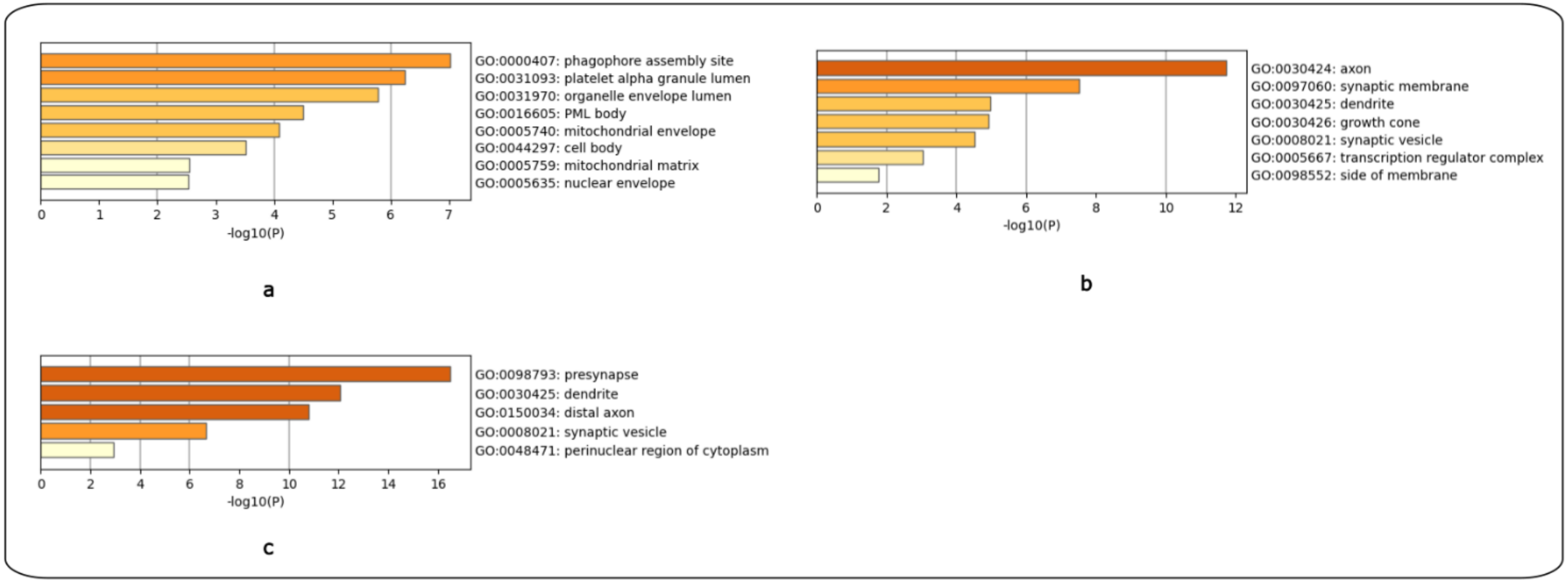

Figure S4. MCODE gene modules GO-Cellular Components analysis using Metascape. (a) MCODE Cluster 1 GO analysis, (b) MCODE Cluster 2 GO and (c) MCODE Cluster 3 GO.

miRNA name	Evidence Code	Disease name	PMID	Description	Causality
hsa-mir-10b	circulation_biomarker_diagnosis_ns	Huntington Diseas	25889241	These results demonstrate that	NO
hsa-mir-125b-1	target gene	Huntington Diseas	21887328	The authors conclude that (i) m	NO
hsa-mir-125b-1	target gene	Huntington Diseas	22048026	Micro RNA -214,-150,-146a and	YES
hsa-mir-125b-2	target gene	Huntington Diseas	21887328	The authors conclude that (i) m	NO
hsa-mir-125b-2	target gene	Huntington Diseas	22048026	Micro RNA -214,-150,-146a and	YES
hsa-mir-128	circulation_biomarker_diagnosis_ns	Huntington Diseas	30359470	hsa-miR-338-3p, hsa-miR-128-3	NO
hsa-mir-128	therapeutic target	Huntington Diseas	24929669	Our studies found that miR-128	NO
hsa-mir-132	other	Huntington Diseas	29858092	Supplemental Treatment for Hu	NO
hsa-mir-137	other	Huntington Diseas	23145961	We also identified several micro	NO
hsa-mir-137	other	Huntington Diseas	23965969	Regulation of huntingtin gene e	NO
hsa-mir-146a	target gene	Huntington Diseas	21887328	The authors conclude that (i) m	NO
hsa-mir-146a	target gene	Huntington Diseas	22048026	Micro RNA -214,-150,-146a and	YES
hsa-mir-146a	tissue_expression_down	Huntington Diseas	26165466	This increase in the expression	NO
hsa-mir-148a	other	Huntington Diseas	23965969	Regulation of huntingtin gene e	NO
hsa-mir-150	target gene	Huntington Diseas	21887328	The authors conclude that (i) m	NO
hsa-mir-150	target gene	Huntington Diseas	22048026	Micro RNA -214,-150,-146a and	YES
hsa-mir-196a	other	Huntington Diseas	23810380	miR-196a ameliorates phenotyp	NO
hsa-mir-196a	other	Huntington Diseas	27631085	miR-196a Ameliorates Cytotoxi	NO
hsa-mir-19a	tissue_expression_down	Huntington Diseas	26165466	This increase in the expression	NO
hsa-mir-200a	therapeutic target	Huntington Diseas	22906125	Altered expression of miR-200a	NO
hsa-mir-200c	therapeutic target	Huntington Diseas	22906125	Altered expression of miR-200a	NO
hsa-mir-214	other	Huntington Diseas	23965969	Regulation of huntingtin gene e	NO
hsa-mir-214	target gene	Huntington Diseas	22048026	Micro RNA -214,-150,-146a and	YES
hsa-mir-214	target gene	Huntington Diseas	26307536	In summary, we have shown th	YES
hsa-mir-22	target gene	Huntington Diseas	23349832	MicroRNA-22 (miR-22) Overexp	YES
hsa-mir-23a	circulation_biomarker_diagnosis_ns	Huntington Diseas	30359470	hsa-miR-338-3p, hsa-miR-128-3	NO
hsa-mir-24	circulation_biomarker_diagnosis_ns	Huntington Diseas	30359470	hsa-miR-338-3p, hsa-miR-128-3	NO
hsa-mir-338	circulation_biomarker_diagnosis_ns	Huntington Diseas	30359470	hsa-miR-338-3p, hsa-miR-128-3	NO
hsa-mir-34a	target gene	Huntington Diseas	29289683	Perturbations in the p53/miR-34	NO
hsa-mir-34b	circulation_biomarker_diagnosis_up	Huntington Diseas	21421997	Hsa-miR-34b is a plasma-stabl	NO

Figure S5. The 20 miRNAs associated with HD.

Table S21. KEGG pathways and miRNA identified using miRPath.

KEGG Pathway Name	p-value	No. miRNAs
Proteoglycans in cancer	0.0000000000179	17
Glycosphingolipid biosynthesis - lacto and neolacto series	0.000000000091	15
ErbB signaling pathway	0.00000000515	17
TGF-beta signaling pathway	0.000000175	17
Prion diseases	0.000000422	11
Adrenergic signaling in cardiomyocytes	0.0000188	17
Neurotrophin signaling pathway	0.0000405	17
FoxO signaling pathway	0.000115133	17
Glioma	0.000171704	17
Adherens junction	0.000175813	16
Renal cell carcinoma	0.000185834	17
Long-term depression	0.000258336	16
MAPK signaling pathway	0.000334537	17
Thyroid hormone signaling pathway	0.000610604	15
Rap1 signaling pathway	0.001418236	17
Endometrial cancer	0.001537697	16
Choline metabolism in cancer	0.001537697	16
Oxytocin signaling pathway	0.001696009	17
Pathways in cancer	0.002100057	17
Chronic myeloid leukemia	0.002100057	17
Hippo signaling pathway	0.002112064	16
Axon guidance	0.002377155	17
Biotin metabolism	0.002557298	3
Glycosaminoglycan biosynthesis - keratan sulfate	0.002557298	8
Gap junction	0.004437047	15
Focal adhesion	0.004446623	17
SNARE interactions in vesicular transport	0.005211951	13
Amphetamine addiction	0.005211951	16
cGMP-PKG signaling pathway	0.005211951	17
cAMP signaling pathway	0.005211951	17
Glycosaminoglycan biosynthesis - chondroitin sulfate / dermatan sulfate	0.005414818	10
Thyroid hormone synthesis	0.005414818	16
Platelet activation	0.005796263	17
Ras signaling pathway	0.005884787	17
Non-small cell lung cancer	0.006779352	17
Insulin secretion	0.007521291	15
mTOR signaling pathway	0.009291295	16
ECM-receptor interaction	0.009722311	17
Long-term potentiation	0.010511377	17

Vascular smooth muscle contraction	0.010995824	16
Hepatitis B	0.011225107	17
Adipocytokine signaling pathway	0.012654222	15
Regulation of actin cytoskeleton	0.012654222	17
Endocrine and other factor-regulated calcium reabsorption	0.01299325	14
Cell cycle	0.01299325	17
Sphingolipid signaling pathway	0.014039474	15
Colorectal cancer	0.014039474	15
Inflammatory mediator regulation of TRP channels	0.015000753	17
Gastric acid secretion	0.015000753	17
PI3K-Akt signaling pathway	0.020727553	17
Estrogen signaling pathway	0.020727553	17
Acute myeloid leukemia	0.02087498	15
Signaling pathways regulating pluripotency of stem cells	0.021444602	17
Prolactin signaling pathway	0.021494327	17
Prostate cancer	0.024491463	17
Thyroid cancer	0.027383025	14
AMPK signaling pathway	0.02758174	16
Protein processing in endoplasmic reticulum	0.02758174	17
Oocyte meiosis	0.030611051	17
Endocytosis	0.035911303	17
Dilated cardiomyopathy	0.036110914	15
Small cell lung cancer	0.037161283	15
Tight junction	0.037161283	17
Wnt signaling pathway	0.039846168	17
Lysine degradation	0.040650489	16
Sphingolipid metabolism	0.04359485	13
Ubiquitin mediated proteolysis	0.045064028	17
GnRH signaling pathway	0.045064028	17
Viral carcinogenesis	0.045064028	17
Phosphatidylinositol signaling system	0.047453049	15
p53 signaling pathway	0.047453049	16

Table S22. KEGG pathways and top 11 miRNAs identifed from the miRNA-gene network.

KEGG Pathway Name	p-value	No. miRNAs
Glycosphingolipid biosynthesis - lacto and neolacto series	4.67E-20	6
Proteoglycans in cancer	0.0000152	7
ErbB signaling pathway	0.000843137	7
Rap1 signaling pathway	0.000843137	7
TGF-beta signaling pathway	0.001034129	7
Gap junction	0.001429867	7
Choline metabolism in cancer	0.001429867	7
Ras signaling pathway	0.00245207	7
Inflammatory mediator regulation of TRP channels	0.00245207	7
Glioma	0.00245207	7
Signaling pathways regulating pluripotency of stem cells	0.003247713	7
mTOR signaling pathway	0.00407108	6
Adherens junction	0.006282367	6
Glycosaminoglycan biosynthesis - keratan sulfate	0.008051398	4
Colorectal cancer	0.009641505	6
Oxytocin signaling pathway	0.010492576	7
Endometrial cancer	0.010540796	7
cGMP-PKG signaling pathway	0.011317129	7
Adrenergic signaling in cardiomyocytes	0.011317129	7
Prolactin signaling pathway	0.011317129	7
FoxO signaling pathway	0.012765416	7
Glycosaminoglycan biosynthesis - chondroitin sulfate / dermatan sulfate	0.013670525	6
MAPK signaling pathway	0.013670525	7
Phosphatidylinositol signaling system	0.016426276	6
Pathways in cancer	0.016426276	7
Non-small cell lung cancer	0.016426276	7
Long-term potentiation	0.017791191	7
Focal adhesion	0.018678183	7
Thyroid cancer	0.019070352	6
Vascular smooth muscle contraction	0.019070352	7
SNARE interactions in vesicular transport	0.03957637	6
Synaptic vesicle cycle	0.03957637	6
Regulation of actin cytoskeleton	0.03957637	7
Hepatitis B	0.04274188	7
Prostate cancer	0.043633467	7
Melanoma	0.048398752	6
